# To Nick or Not to Nick: Comparison of I-SceI Single- and Double-Strand Break-Induced Recombination in Yeast and Human Cells

**DOI:** 10.1371/journal.pone.0088840

**Published:** 2014-02-18

**Authors:** Samantha S. Katz, Frederick S. Gimble, Francesca Storici

**Affiliations:** 1 School of Biology, Georgia Institute of Technology, Atlanta, Georgia, United States of America; 2 Department of Biochemistry, Purdue University, West Lafayette, Indiana, United States of America; Universita' di Milano, Italy

## Abstract

Genetic modification of a chromosomal locus to replace an existing dysfunctional allele with a corrected sequence can be accomplished through targeted gene correction using the cell's homologous recombination (HR) machinery. Gene targeting is stimulated by generation of a DNA double-strand break (DSB) at or near the site of correction, but repair of the break via non-homologous end-joining without using the homologous template can lead to deleterious genomic changes such as in/del mutations, or chromosomal rearrangements. By contrast, generation of a DNA single-strand break (SSB), or nick, can stimulate gene correction without the problems of DSB repair because the uncut DNA strand acts as a template to permit healing without alteration of genetic material. Here, we examine the ability of a nicking variant of the I-SceI endonuclease (K223I I-SceI) to stimulate gene targeting in yeast *Saccharomyces cerevisiae* and in human embryonic kidney (HEK-293) cells. K223I I-SceI is proficient in both yeast and human cells and promotes gene correction up to 12-fold. We show that K223I I-SceI-driven recombination follows a different mechanism than wild-type I-SceI-driven recombination, thus indicating that the initial DNA break that stimulates recombination is not a low-level DSB but a nick. We also demonstrate that K223I I-SceI efficiently elevates gene targeting at loci distant from the break site in yeast cells. These findings establish the capability of the I-SceI nickase to enhance recombination in yeast and human cells, strengthening the notion that nicking enzymes could be effective tools in gene correction strategies for applications in molecular biology, biotechnology, and gene therapy.

## Introduction

Gene targeting is a technique that is used to genetically modify cellular DNA. This form of genetic manipulation relies on HR to facilitate exchange of DNA sequences between the targeted chromosomal locus and a homologous template containing the desired change(s) [1]–[5]. Because many genetic disorders are caused by a single point mutation, gene targeting has developed as a powerful technique for repairing a defective DNA sequence by targeted replacement with a functional copy [4]–[6]. However, because HR is inefficient in many organisms, a site-specific DSB is often introduced in the vicinity of the targeted genomic locus in order to stimulate repair [7]–[9]. Numerous studies have demonstrated this activity using meganucleases (homing endonucleases), such as I-SceI, I-AniI and HO (F-SceII), which naturally have large cognate recognition sequences [7]–[11]. Additionally, artificial endonucleases, such as zinc finger nucleases (ZFNs) and transcription activator-like effector nucleases (TALENs), which can be custom-designed to cleave at specific DNA sequences of interest, have widely been employed for their targeted gene correction potential [12]–[16]. Most recently, clustered, regularly interspaced, short palindromic repeats (CRISPR) associated (Cas) systems, which use an RNA guide to target a specific DNA sequence for cleavage, have shown great promise for further advancing gene targeting strategies [17]–[19]. However, while a DSB can efficiently stimulate HR up to 10,000-fold [7]–[10], [20], the competing non-homologous end-joining (NHEJ) pathway for DSB repair is often favored, especially in human cells, and poses a major safety problem for gene targeting strategies, particularly for gene therapy applications, because it frequently leads to in/dels or chromosomal rearrangements [21]–[23]. Thus, it is important to develop approaches other than those that use a DSB to stimulate HR in order to provide a safer alternative for gene correction.

Another means to stimulate HR for targeted gene correction is through the generation of a site-directed SSB, or nick. As opposed to a DNA double-strand endonuclease, a nickase cleaves a single DNA strand. Recent work has shown that an SSB leads to less off-site targeting than a DSB [24], [25]. Studies in yeast and human cells have demonstrated that an SSB can facilitate gene targeting. These involve employing a bona fide nickase, such as Gene II from the bacteriophage f1 [20], [26], using nicking variants of natural meganucleases, such as I-AniI [25], [27], designing nickases with zinc finger binding domains [28]–[30], and, more recently, employing a CRISPR/Cas system [17], [18]. However, questions remain concerning the kinetics of SSB-driven gene targeting. For example, though generation of an SSB has been followed by stimulation of HR, it is unknown whether this lesion itself or its successive conversion into a DSB triggers the repair pathway. Also unclear is whether an SSB can initiate recombination at a genomic position located distant from the site of the break, or whether repair is only confined to the window adjacent to the lesion.

Here, we show that an I-SceI-derived SSB increases targeted gene correction in the yeast *S. cerevisiae* and in human cells. A nicking variant (K223I I-SceI) of the wild-type I-SceI protein was generated which demonstrated *in vitro* activity different from wild-type I-SceI [31]. A lysine to isoleucine substitution at residue 223 was shown to largely abolish enzymatic cleavage of one strand of the 18-bp cognate I-SceI recognition site [31]. However, the capacity of K223I I-SceI to increase gene targeting *in vivo* has not been reported yet. This study reveals that K223I I-SceI acts as a nickase *in vivo* and demonstrates that K223I I-SceI stimulates gene correction by a different mechanism than wild-type I-SceI. Genetic controls, cell cycle activity, and preference for different repairing molecules were assayed in yeast in this study. Moreover, we found that K223I I-SceI can increase gene targeting in human cells both at a target plasmid locus as well as on the chromosome. These results demonstrate the capacity of the I-SceI nickase to stimulate HR in different genetic systems and provide new insights on the functions and mechanism of nicking proteins for gene targeting.

## Results

### Cleavage activity *in vitro* of the I-SceI nicking protein variant

**Figure 1:**
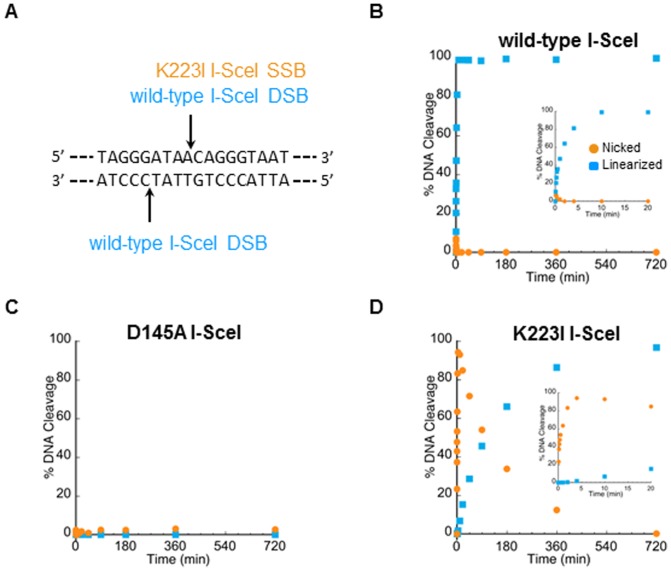
DNA cleavage activities of the wild-type, D145A, and K223I I-SceI proteins. (A) Scheme of the 18-bp I-SceI recognition sequence showing the cleavage positions of wild-type I-SceI and K223I I-SceI. Supercoiled pBS-I-SceI (E/H) plasmid DNA was incubated with (B) wild-type I-SceI, (C) D145A I-SceI mutant, or (D) K223I I-SceI mutant for various lengths of time and the amounts of the nicked open circle (orange circles) and linear (blue squares) reaction product DNAs were plotted as a function of time. Data points represent the average values of two experiments. Insets show the same data immediately following initiation of the reactions.

To determine the efficacy and the mechanism of SSB-driven gene targeting, we chose a nickase whose activity could be directly compared to variant forms of the same enzyme that do not cleave DNA or that cleave both DNA strands. These different variants of the double-strand endonuclease I-SceI homing enzyme were generated previously [31]. K223I I-SceI contains an amino acid substitution that borders the catalytic center and rapidly cleaves one specific DNA strand at the cognate 18-bp I-SceI recognition sequence but not the other (**Figure 1A**) without producing a hairpin product [31]. D145A I-SceI is a variant enzyme that contains an alanine substitution of an essential active site residue and is completely defective in DNA cleavage activity [31]. To compare the *in vitro* cleavage activities of the wild-type, K223I, and D145A I-SceI proteins, a supercoiled plasmid (pBS-I-SceI (E/H)) containing a single I-SceI recognition sequence was incubated with each of these proteins. Within 20 minutes, both DNA strands of the plasmid were completely cleaved by the wild-type I-SceI protein to yield the linearized product (**Figure 1B**), but the D145A I-SceI variant failed to cleave the plasmid DNA even after 12 hours (**Figure 1C**). The K223I I-SceI variant produced a nicked open circular product within 20 minutes which was slowly converted into the linearized form over many hours (**Figure 1D**), indicating that the enzyme efficiently generates an SSB but not a DSB. K223I nicking rate is approximately the same as the double-strand cleavage rate by wild-type I-SceI. We estimate that nicking of the DNA by the K223I I-SceI variant occurs approximately 180-fold faster than linearization.

### A K223I-I-SceI-induced break stimulates homologous recombination in yeast

To test the *in vivo* capacity of I-SceI SSB-induced recombination in yeast, we generated plasmids expressing the wild-type, K223I, or D145A I-SceI proteins under the control of the galactose-inducible *GAL1* promoter. We compared the frequency of gene correction obtained by recombination after expression of wild-type I-SceI or K223I I-SceI with the frequency of correction obtained after expression of D145A I-SceI, which served as a non-break control. Haploid yeast strains SAS-74 and SAS-75, SAS-77 and SAS-149, SAS-142 and SAS-143 (listed in **Table S1**) were constructed that contained the plasmids expressing the wild-type (pAG7-wild-type-I-SceI), K223I (pAG7-K223I), or D145A (pAG7-D145A) I-SceI variants, respectively.

**Figure 2:**
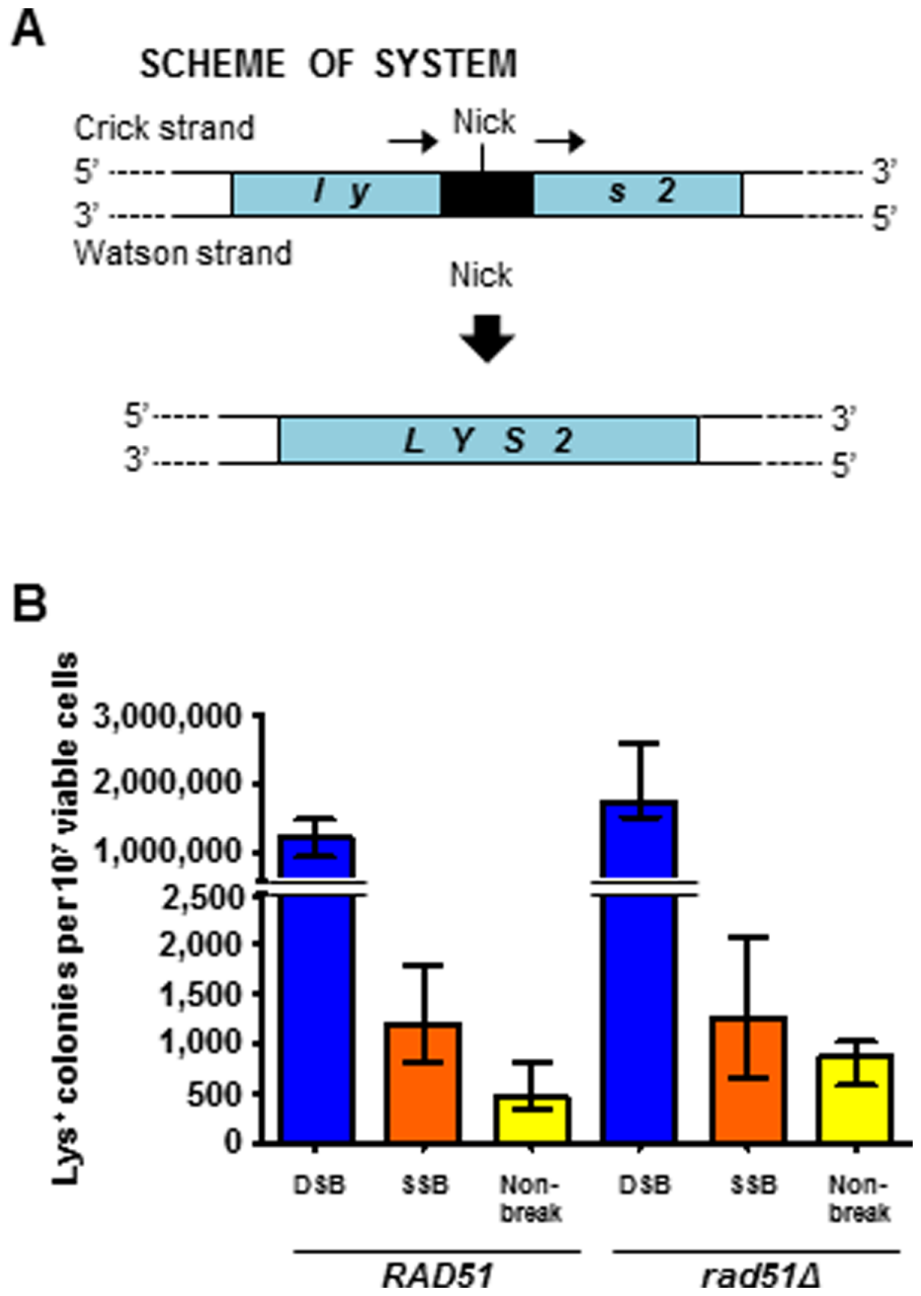
An I-SceI K223I break stimulates HR between direct repeats in yeast. (A) Scheme showing disrupted yeast *lys2* chromosomal locus containing the I-SceI recognition sequence (black box) within 90-bp direct repeats (small arrows). The position of the SSB is indicated (“Nick”). DNA strands are identified as “Crick” and “Watson” according to the *Saccharomyces cerevisiae* Genome Database (SGD). (B) Frequency of Lys^+^ recombinants following expression of wild-type I-SceI (dark blue bars labeled “DSB”), K223I I-SceI (orange bars labeled “SSB”), or D145A I-SceI (yellow bars labeled “Non-break”) in *RAD51* wild-type (left) or *rad51*-null mutant (right) strains are presented as the median with range (n≥11). For the specific numerical values see Table S3A. Wild-type I-SceI strains used: SAS-74 and SAS-75 (*RAD51*), and SAS-174 and SAS-175 (*rad51*Δ). K223I I-SceI strains used: SAS-77 and SAS-149 (*RAD51*), and SAS-176 and SAS-177 (*rad51*Δ). D145A I-SceI strains used: SAS-142 and SAS-143 (*RAD51*), and SAS-178 and SAS-179 (*rad51*Δ).

We first examined I-SceI-stimulated recombination between direct repeats (**Figure 2A**) following expression of the different I-SceI variants. In the assay strain, the *LYS2* genomic locus is disrupted and inactivated by an I-SceI recognition site that is flanked by direct repeats of a 90-bp *LYS2* sequence. Generation of a DSB at the I-SceI site triggers the single-strand annealing (SSA) pathway in which 5′ to 3′ resection of both DNA ends is followed by annealing of the exposed single-stranded repeat regions and filling of the gaps, ultimately leading to reconstitution of a functional *LYS2* gene [32]. Following expression of wild-type I-SceI and generation of the DSB, recombination between the repeats was stimulated more than 2,000-fold (p<0.0001). While the frequency was much lower following expression of K223I I-SceI relative to wild-type I-SceI, there was a small but significant 2.4-fold increase over the non-break control (p<0.0001) (**Figure 2B**, left).

**Figure 3:**
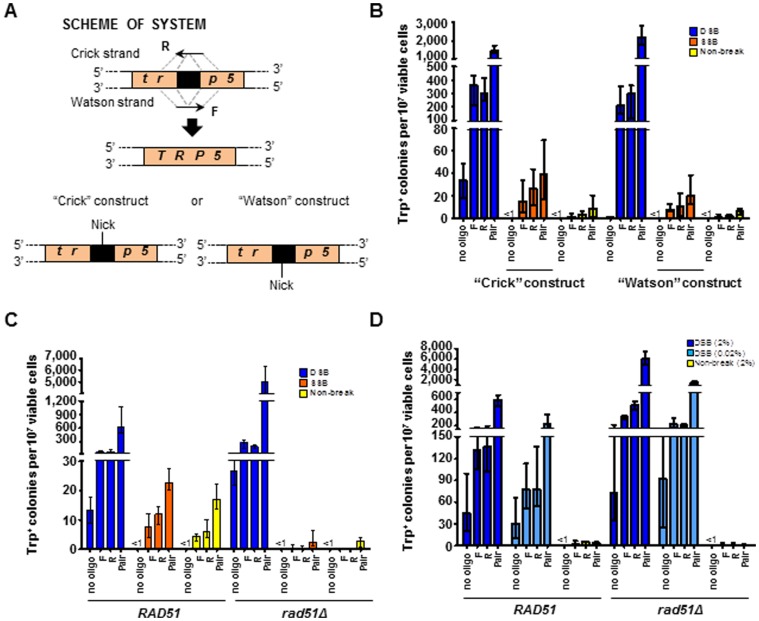
A K223I I-SceI break stimulates gene correction by oligonucleotides in yeast. (A) Scheme showing disrupted yeast *trp5* chromosomal locus containing the I-SceI recognition sequence (black box). The position of the SSB is indicated (“Nick”) for the “Crick” and “Watson” constructs. Dashed gray lines indicate the complementarity between the F oligonucleotide and the antisense strand of the targeted gene, and between the R oligonucleotide and the sense strand of the targeted gene. (B–D) Frequencies of Trp^+^ transformants following expression of wild-type I-SceI (dark blue bars labeled “DSB”), K223I (orange bars labeled “SSB”), or D145A (yellow bars labeled “Non-break”) using either of the single or the pair of oligonucleotides to repair the break. All data are presented as the median with range (n≥5). For the specific numerical values see Table S3B-D. (B) Gene correction frequencies by oligonucleotides when an SSB is generated on the “Crick” (left) or “Watson” (right) chromosomal strand. (C) Frequency of transformants in *RAD51* (left) or *rad51* null mutant (right) strains when the SSB is generated on the “Crick” strand. (D) Frequency of transformants following expression of wild-type I-SceI in *RAD51* (left) or *rad51* null mutant (right) strains with final galactose concentrations of 2% (dark blue bars labeled “DSB (2%)”) or 0.02% (light blue bars labeled “DSB (0.02%)”). Wild-type I-SceI strains used: SAS-227 and SAS-228 (“Crick” and *RAD51*), SAS-281 and SAS-282 (“Watson”), and SAS-235 and SAS-236 (*rad51*Δ). K223I I-SceI strains used: SAS-229 and SAS-230 (“Crick” and *RAD51*), SAS-283 and SAS-284 (“Watson”), and SAS-237 and SAS-238 (*rad51*Δ). D145A I-SceI strains used: SAS-231 and SAS-232 (“Crick” and *RAD51*), SAS-285 and SAS-286 (“Watson”), and SAS-239 and SAS-240 (*rad51*Δ).

Next, we tested gene correction using DNA oligonucleotides following expression of the different I-SceI variants, in an assay strain in which the genomic *TRP5* locus has been disrupted by insertion of the I-SceI recognition site in opposite orientations (**Figure 3A**). In strains derived from SAS-193, K223I I-SceI can only generate a nick on the “Crick” strand (SAS-229 and SAS-230), while in strains derived from SAS-278, K223 I-SceI can only generate a nick on the “Watson” strand (SAS-283 and SAS-284) (**Table S1**). DNA oligonucleotides 80-bp in length were designed with homology to either side of the *trp5* disruption site such that they could restore the sequence of the *TRP5* gene if used as a repair template. Previously, it was shown that oligonucleotides can efficiently transfer genetic modifications to genomic DNA in yeast following generation of a DSB at the targeting locus [10]. Similarly, after expression of wild-type I-SceI in strains SAS-227 and SAS-228 a DSB was generated which was repaired using oligonucleotides (TRP5.80F (F), corresponding to the sense strand of the gene, and TRP5.80R (R), representing the antisense strand) designed to restore the sequence of the disrupted *trp5* locus (**Table S2**). While Trp^+^ colonies were detected following generation of the DSB even without oligonucleotides, the sequence of the *TRP5* locus in these colonies differs from those appearing following recombination with the oligonucleotides and these numbers were subtracted from the counts with oligonucleotides prior to statistical analysis. Specifically, 2 random Trp^+^ clones deriving from no-oligonucleotide transformation in cells expressing wild-type I-SceI were tested for the presence of the BamHI site. Following PCR amplification of the *TRP5* locus using primers TRP5.80F and TRP5.80R, and restriction digestion by BamHI, 2/2 had the PCR product uncut. Differently, 10/10 clones derived from F plus R oligonucleotide transformation in cells expressing wild-type I-SceI or K223I I-SceI had the PCR product cut by BamHI showing that the *trp5* allele was repaired by the oligonucleotide sequence (**Figure S1**). Gene correction following DSB induction was efficient for the complementary pair compared to the non-break control (160-fold increase: p = 0.0022) as well as for the single-stranded F or R oligonucleotide compared to the non-break control (190-fold increase for F: p = 0.0022, or 80-fold increase for R: p = 0.0022, respectively; **Figure 3B**, left). Similarly, when expression of K223I I-SceI in strains SAS-229 and SAS-230 was used to introduce a break, an increase in recombination frequency was observed following transformation with oligonucleotides F, R, and the F+R pair (up to 9-fold increase for F: p = 0.0022; 8.2-fold for R: p = 0.0022; and 4.5-fold for pair: p = 0.0087, respectively). Recombination using the 80-mers after K223I I-SceI expression was 3-10% as efficient relative to that observed after expression of wild-type I-SceI. Similar results were obtained using the strains containing the “Watson” construct (**Figure 3B**, right). While fewer colonies arose following generation of the DSB in the “no oligo” control in this construct, likely due to the different distribution of stop codons between the constructs upon end joining, those Trp^+^ colonies which were detected were subtracted from values with oligonucleotides prior to statistical analysis. Additionally, there was no significant strand bias for gene correction by oligonucleotides following expression of wild-type I-SceI or K223I I-SceI in strains containing either the “Crick” (p≥0.1797) (**Figure 3B**, left) or the “Watson” (p≥0.3776) (**Figure 3B**, right) constructs as determined by comparing the frequencies of recombination using the F versus R oligonucleotide.

### Gene correction stimulated by a K223I I-SceI break in yeast requires Rad51 function

Our *in vitro* experiment demonstrated that K223I I-SceI efficiently generates a nick but also forms a DSB at the I-SceI recognition site following prolonged incubation with the substrate (**Figure 1D**). In order to determine if the capacity of K223I I-SceI to stimulate gene correction was directly due to its nicking activity rather than its low-level DSB activity, we first examined if K223I I-SceI-driven recombination was dependent on Rad51 function. For this purpose, we compared the frequencies of recombination between direct repeats and of gene correction by oligonucleotides following expression of wild-type I-SceI, K223I I-SceI or D145A I-SceI using strains in which *RAD51* was deleted (SAS-174, SAS-175, SAS-176, SAS-177, SAS-178 and SAS-179 for the test with direct repeat recombination; SAS-235, SAS-236, SAS-237, SAS-238, SAS-239, and SAS-240, for the test of oligonucleotide-driven gene correction; **Table S1**). Previous work showed that recombination between direct repeats *via* SSA doesn't require Rad51 [32], [33] and that DSB repair by single-stranded oligonucleotides is also independent of Rad51 [34]. These studies suggest that in both direct repeat and oligonucleotide-mediated repair the deletion of Rad51 prevented the search for a homologous sequence on a sister chromatid, thereby facilitating repair of the DSB using the repeated sequence or oligonucleotide. In agreement with work published previously by others [32], the frequency of Lys^+^ colonies increased 1.5-fold (p<0.0001) in the *rad51*Δ mutant background compared to *RAD51* cells following expression of wild-type I-SceI (**Figure 2B**, right), and a 1.8-fold increase (p = 0.0001) was observed for the non-break control, due to the short length of the closely-spaced direct repeats which could easily facilitate alignment of the homologous segments without an extensive homology search [33], [35]. By contrast, we did not detect significant difference in gene correction frequency with the K223I I-SceI break (p = 0.4302). Similarly to what was observed for recombination between direct repeats, we measured a 2.9- to 8.1-fold increase in Trp^+^ colonies following transformation with the single or the pair of oligonucleotides after DSB induction (p≤0.0043) in *rad51*Δ cells compared to *RAD51* cells (**Figure 3C**, right). Remarkably, we observed a 9.1- to 34-fold decrease in gene correction frequency using the single or complementary pair of oligonucleotides (p = 0.0022 for F, R and pair) after expression of K223I I-SceI in *rad51*Δ cells (**Figure 3C**, right).

**Figure 4:**
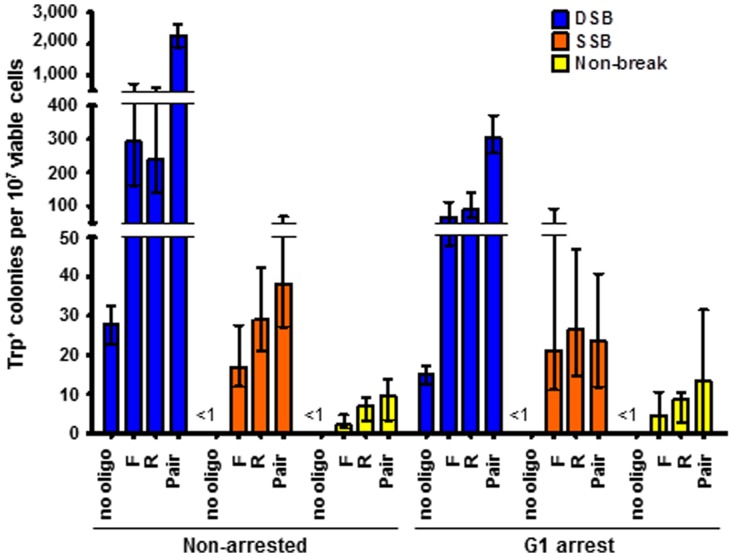
A nick occurring in asynchronous or G1 arrested cells stimulates gene correction by oligonucleotides equally efficiently. Shown are frequency of Trp^+^ transformants by oligonucleotides when cells were asynchronous (left) or arrested in G1 (right) at the time of wild-type-I-SceI or K223I I-SceI breakage prior to oligonucleotide transformation and when the SSB is generated on the “Crick” strand. All data are presented as the median with range (n≥5). For the specific numerical values see Table S3E. Wild-type I-SceI strains used: SAS-227 and SAS-228. K223I I-SceI strains used: SAS-229 and SAS-230. D145A I-SceI strains used: SAS-231 and SAS-232.

To demonstrate that the K223I I-SceI nicking activity, rather than its low DSB activity, is responsible for the observed Rad51-dependent gene correction by oligonucleotides, we determined if gene correction stimulated by wild-type I-SceI was dependent on Rad51 under conditions of low I-SceI expression. We adjusted the galactose concentration (0.02%) in the media to express sufficient wild-type I-SceI to stimulate gene correction 10-fold (p = 0.0043) or 4.2-fold (p = 0.0022) by the F or R oligonucleotides, respectively, above the level obtained when D145A I-SceI is expressed (after subtraction of the background). This level of gene targeting stimulation is approximately equal to that observed using the F and R oligonucleotides when K223I I-SceI is expressed in *RAD51* cells in media containing 2% galactose (**Figure 3D**, left, light blue bars relative to yellow bars after background subtraction are compared to orange bars relative to yellow bars presented in **Figure 3B** left). Even at these lower frequencies of gene correction by using the F and R oligonucleotides, deletion of *RAD51* still stimulated recombination (3.6-fold increase for F, p = 0.0152, and 2.9-fold increase for R, p = 0.0411, respectively) with wild-type I-SceI as compared to D145A control (**Figure 3D**, right). We also note that the background of Trp^+^ cells obtained with the no-oligonucleotide control is substantial only when wild-type I-SceI is expressed, even in low galactose concentration, and the nucleotide sequence of *TRP5* in these cells differs from the sequence when an oligonucleotide is used to repair the lesion (**Figure S1**), suggesting an end joining mechanism of DSB repair in the absence of a repair template. Differently, the background of Trp^+^ cells for the no-oligonucleotide control was always <1 per 10^7^ viable cells following expression of K223I I-SceI (**Figure 3B-C** and see also **Figure 4**), indicating that the broken *trp5* marker cannot be repaired by end joining of the broken ends. These data are in line with the notion that only the break caused by wild-type I-SceI and not the one caused by K223I I-SceI is a DSB, which can be repaired by NHEJ. Overall, these results are strongly consistent with direct SSB-stimulated gene correction rather than gene correction promoted by low DSB levels following expression of K223I I-SceI. These findings indicate that the K223I I-SceI break is different from the one generated by wild-type I-SceI, and we conclude that the initial gene correction-stimulating lesion is an SSB.

### A K223I I-SceI SSB stimulates gene correction to the same extent in actively growing or G1-arrested cells

We next examined the frequency of gene correction by oligonucleotides after induction of the nick in cells arrested in the G1 phase of the cell cycle. Because gene correction stimulated by a DSB is low, if the DSB occurs in the G1 phase of the cell cycle due to the lack of resection [36], we tested if this was also the case for gene correction stimulated by the K223I I-SceI SSB. We arrested the *trp5* strains expressing wild-type, K223I or D145A I-SceI (SAS-227, SAS-228, SAS-229, SAS-230, SAS-231, and SAS-232, respectively) in G1 using yeast alpha factor (see Methods), and we then transformed these strains with the TRP5.80F and TRP5.80R oligonucleotides. Frequencies of Trp^+^ colonies significantly decreased up to 7.5-fold in the SAS-227 and SAS-228 strains in cells transformed after G1-arrest expressing wild-type I SceI (p = 0.0022 for F, p = 0.0087 for R, and p = 0.0043 for the pair). By contrast, gene correction frequencies were unaffected in the SAS-229 and SAS-230 strains expressing K223I I-SceI when cells were arrested in G1 prior to transformation compared to non-arrested cells (p =  0.5745 for F, p = 0.5887 for R, and p = 0.0649 for the pair) (**Figure 4**). We also note that cells subjected to the transformation procedure but without oligonucleotides can become Trp^+^ only if they express wild-type I-SceI but not K223I I-SceI both if arrested in G1 or not, similarly to what was observed in experiments shown in **Figure 3B, C and D**, suggesting repair by NHEJ. These data support the evidence that K223I I-SceI triggers gene correction using a nick and not a low-level DSB as the recombination-stimulating lesion.

### A K223I I-SceI SSB stimulates HR at the site of the break in human cells

**Figure 5:**
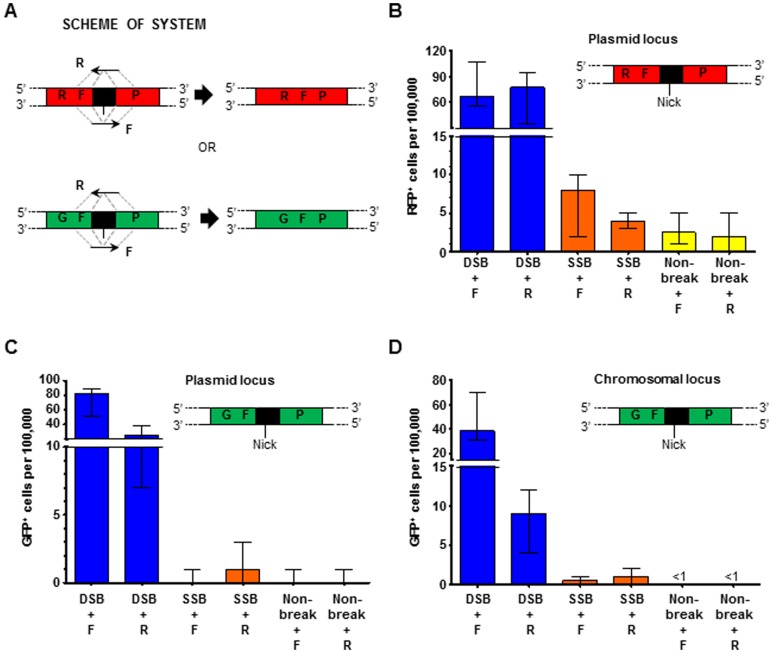
K223I I-SceI SSB can stimulate recombination in human cells. (A) Scheme showing disrupted RFP plasmid locus and disrupted GFP plasmid or chromosomal locus containing the I-SceI recognition sequence (black box). The position of the SSB is indicated by a black line. Dashed gray lines indicate the complementarity between the F oligonucleotide and the antisense strand of the targeted gene, and between the R oligonucleotide and the sense strand of the targeted gene. (B–D) Frequency of fluorescent cells following expression of wild-type I-SceI (dark blue bars labeled “DSB”), K223I I-SceI (orange bars labeled “SSB”), or D145A I-SceI (yellow bars labeled “Non-break”) using either of the single oligonucleotides to repair the break. All data are presented as the median with range. For the specific numerical values see Table S3F-H. (B) Recombination at the RFP target plasmid locus (n = 6). (C) Recombination at the GFP target plasmid locus (n = 9). (D) Recombination at the GFP target chromosomal locus (n≥8).

In order to examine if a K223I I-SceI SSB could stimulate gene correction in human cells, we utilized plasmids expressing wild-type, K223I, or D145A I-SceI under a strong CMV/CBA hybrid promoter. The plasmids expressing wild-type, K223I, or D145A I-SceI, a vector containing the I-SceI site within a disrupted target locus, and 80-bp synthetic DNA oligonucleotides were co-transfected into HEK-293 cells following procedures previously described [37]. The target plasmid loci, GFP or DsRed2 (referred to as RFP), were disrupted by an insert which includes two stop codons and the 18-bp recognition sequence for I-SceI. F and R oligonucleotides (GFP.80F and GFP80.R for GFP or DsRed2.80F and DsRed2.80R for RFP) were designed to restore the sequence of the disrupted gene, yielding GFP^+^ or RFP^+^ cells, depending on the marker (**Table S2**). For both the GFP and the RFP constructs, the F oligonucleotide corresponds to the sense strand of the gene and the R oligonucleotide represents the antisense sequence (**Figure 5A**). The frequency of GFP^+^ or RFP^+^ cells obtained after gene correction by the oligonucleotides was determined by flow cytometric analysis 5–8 days post-transfection.

The plasmid containing the disrupted RFP marker (pGRdis) was co-transfected into HEK-293 cells along with the RFP repair oligo, F or R, and an I-SceI expression vector expressing wild-type, K223I or D145A I-SceI. Following generation of the I-SceI DSB by wild-type I-SceI, frequencies of RFP^+^ cells significantly increased using either the F or R oligonucleotide (27-fold increase for F, p = 0.0050, and 39-fold increase for R, p = 0.0048, respectively) over the non-break control. No strand bias targeting was observed at the RFP locus for repair of the lesion generated by either wild-type or K223I I-SceI (p = 0.6991 and p = 0.3743, respectively). While there was no significant increase relative to the non-break control using F following expression of K223I I-SceI (p = 0.1215), there was an increase in gene correction frequencies at RFP using the R oligonucleotide following expression of K223I I-SceI (2-fold (p = 0.0423) (**Figure 5B**)). At this plasmid locus, K223I I-SceI was ∼5% as efficient as the wild-type protein at stimulating recombination. At the RFP plasmid locus, higher than expected numbers of RFP^+^ cells (per 100,000 cells median = 5 (5–5) for F and median = 3 (2–6) for R) were detected for the negative control in which the target locus and either repairing oligonucleotide were provided (no-enzyme control, data not shown). All other negative controls (with no oligonucleotides or with any of the oligonucleotides and no plasmid expressing I-SceI) produced <1 fluorescent cell per 100,000 cells (<0.25–0.5, data not shown). The plasmid containing the disrupted GFP marker (pA658) was co-transfected into cells together with the GFP repair oligonucleotides F or R, and an I-SceI expression vector (pSce, pSce-K223I, or pSce-D145A). As expected, following expression of wild-type I-SceI, frequencies of GFP^+^ cells significantly increased using the F or R oligonucleotide (674-fold increase, p<0.0001, and 228-fold increase, p<0.0001, respectively) relative to the non-break control. No increase was observed following expression of K223I I-SceI using the oligonucleotide F to repair the lesion (p>0.9999) but there was a significant 12-fold increase with R (p = 0.0078) relative to the non-break control (**Figure 5C**). At this plasmid locus, K223I I-SceI was ∼5% as efficient as the wild-type protein. All negative controls produced <1 fluorescent cell per 100,000 cells (<0.33–1).

We then examined repair on the chromosome in human cells. The cell line 293/A658 is a monoclonal modified HEK-293 cell line in which a stably integrated copy of the same disrupted GFP sequence used for our plasmid assay was randomly introduced into the genome [12]. We transfected 293/A658 cells with the plasmid expressing wild-type I-SceI, K223I I-SceI, or D145A I-SceI along with the GFP repair oligonucleotide F or R, and measured fluorescence by flow cytometry approximately 8 days later (**Figure 5D**). Following expression of wild-type I-SceI and co-transfection of the repair oligo, the DSB stimulated recombination using F or R relative to the non-break control (330-fold increase, p = 0.0002, and 77-fold increase, p<0.0001, respectively). Similar to the findings observed at the GFP plasmid locus, no increase was observed for repair of the K223I I-SceI lesion with the F oligonucleotide (p = 0.0769) but there was a significant 12-fold increase with the R oligonucleotide (p = 0.0004) compared to the non-break control. On the chromosome at this target GFP locus, the K223I I-SceI lesion was ∼16% as efficient as the DSB at stimulating recombination. All negative controls produced <1 fluorescent cell per 100,000 cells (<0.14–1). Interestingly, at the GFP plasmid and chromosomal loci there was a bias targeting in favor of the F oligonucleotide for repair of the wild-type I-SceI DSB compared to the R oligonucleotide (p<0.0001 in both cases) while the R oligonucleotide was favored for repair of the K223I I-SceI lesion (p = 0.0078 and p = 0.0334, respectively) (**Figure 5C and D**, and see also **Table S3G**), suggesting that K223I I-SceI stimulates recombination by a different mechanism from wild-type I-SceI also in human cells.

### A K223I I-SceI SSB stimulates HR at distant loci in yeast

**Figure 6:**
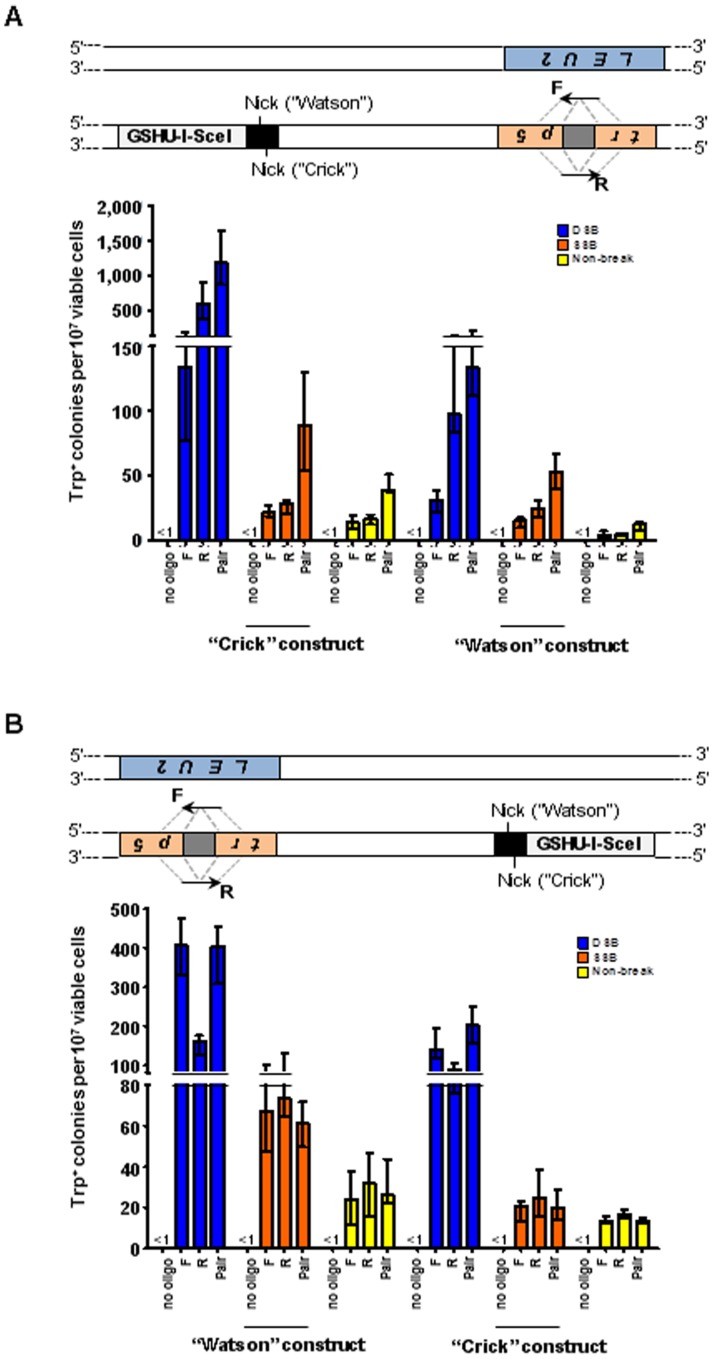
Gene targeting distant from the I-SceI DSB or SSB in yeast cells. Depicted above each graph in (A) and (B) are schemes of the two copies of chromosome VII of diploid yeast cells, one in which *TRP5* has been replaced by *LEU2*, and another in which *TRP5* has been disrupted by a 31-bp insertion (gray box), which does not contain an I-SceI cut site, and which also contains the GSHU-I-SceI cassette with the I-SceI site (black box) inserted 10 kb upstream or downstream of the disrupted *trp5*. Dashed gray lines indicate the complementarity between the F oligonucleotide and the antisense strand of the targeted gene, and between the R oligonucleotide and the sense strand of the targeted gene. The position of the SSB is indicated (“Nick”) for the “Watson” and “Crick” constructs. Frequencies of Trp^+^ transformants following expression of wild-type I-SceI (dark blue bars labeled “DSB”), K223I I-SceI (orange bars labeled “SSB”), or D145A I-SceI (yellow bars labeled “Non-break”) using either of the single oligonucleotides or the pair of oligonucleotides to repair the break. All data are presented as the median with range (n≥5). For the specific numerical values see Table S3I,J. (A) Frequencies of transformants when an SSB is generated on the “Crick” (left) or “Watson” (right) chromosomal strand 10 kb upstream from the *trp5* locus. Wild-type I-SceI strains used: SAS-150 and SAS-151 (“Crick”), and SAS-215 and SAS-217 (“Watson”). K223I I-SceI strains used: SAS-162 and SAS-163 (“Crick”), and SAS-207 and SAS-209 (“Watson”). D145A I-SceI strains used: SAS-166 and SAS-167 (“Crick”), and SAS-211 and SAS-213 (“Watson”). (B) Frequencies of transformants when an SSB is generated on the top (“Watson”, left) or bottom (“Crick”, right) chromosomal strand 10 kb downstream from the *trp5* locus. Wild-type I-SceI strains used: SAS-152 and SAS-153 (“Watson”), and SAS-272 and SAS-274 (“Crick”). K223I I-SceI strains used: SAS-154 and SAS-156 (“Watson”), and SAS-219 and SAS-221 (“Crick”). D145A I-SceI strains used: SAS-158 and SAS-160 (“Watson”), and SAS-251 and SAS-253 (“Crick”).

Previously it was shown in yeast that an I-SceI DSB can stimulate HR at an upstream or downstream locus distant from the break site [34]. The work by Storici *et al*., 2006, showed that DNA ends that can be resected (5′ to 3′ resection) expose long single-stranded regions, and if targeted for gene correction by single-stranded oligonucleotides, strongly favor targeting by the oligonucleotide complementary to the non-resected DNA strands [34]. We sought to determine if an I-SceI SSB generated several kb distant from the target genomic locus could also stimulate HR at that target locus and if any strand bias could be observed. For this purpose, we generated diploid yeast strains in which cassettes containing the wild-type, K223I, or D145A I-SceI genes regulated by the inducible *GAL1* promoter, along with the 18-bp I-SceI recognition site, were integrated approximately 10 kb upstream or downstream from a *trp5* locus, which was disrupted by a 31-bp insert [34] (**Figure 6A top** and **Figure 6B top**). In the upstream and downstream constructs, the I-SceI site was integrated in both orientations at the same chromosomal position such that the SSB is generated on the top (“Watson”) or bottom (“Crick”) chromosomal strand. The TRP5.80F (F) and TRP5.80R (R) oligonucleotides were used to repair the *trp5* locus in these systems, while the break at the distant I-SceI site was repaired by the homologous chromosome.

When the cassette was located upstream, wild-type I-SceI stimulated recombination 7.1- to 35-fold with the F, R, and complementary pair of oligonucleotides at the distant *trp5* locus (p = 0.0286 for F, R, and the complementary pair in both the “Crick” and “Watson” constructs), confirming previous data [34] (**Figure 6A** and **Table S3I**). Remarkably, the K223I I-SceI protein stimulated recombination 1.7- to 5.2-fold (p = 0.0286 for R and the complementary pair in the “Crick” construct and for F, R, and the complementary pair in the “Watson” construct) using either of the single or the complementary pair of oligonucleotides except with the F oligonucleotide in the “Crick” construct (p = 0.0571) (**Figure 6A** and **Table S3I**). Our previous data indicate that a DSB stimulates gene targeting by oligonucleotides at a locus distant from the DSB in a strand bias manner. Specifically, the oligonucleotide that is complementary to 3′ strand from the DSB is more efficient at distant gene correction that the oligonucleotide that is complementary to the 5′ strand of DNA, because this DNA strand is resected and thus cannot provide a target for the complementary oligonucleotide [34]. As expected, strand biased targeting involving the R oligonucleotide was observed following generation of the DSB upstream of the targeting locus (p = 0.0286), but no strand bias was observed involving the SSB in favor of either the F or R oligonucleotide (p≥0.0571) (**Figure 6A**). When the cassette was located downstream of the targeting locus, the DSB stimulated recombination 5.1- to 17-fold with the F, R, and complementary pair of oligonucleotides (p = 0.0286 for F, R, and the complementary pair in both the “Watson” and “Crick” constructs) (**Figure 6B** and **Table S3J**). The SSB stimulated recombination 2.3- to 2.8-fold with the single and complementary pair of oligonucleotides using the “Watson” construct (p = 0.0286 for F, R, and the complementary pair), but no increase in transformants was observed using the “Crick” construct (p≥0.0571), indicating that stimulation of gene correction following generation of a distant SSB may vary depending on location within the chromosome relative to the targeted locus. The difference in gene correction frequency obtained when the I-SceI site is simply inverted in both the upstream and downstream constructs does not have an evident explanation. Because the I-SceI site is asymmetric, this effect may be due to sequence-dependent processing of the broken ends following I-SceI cleavage. Strand biased targeting in favor of the F oligonucleotide was observed after induction of the DSB downstream of the targeting site (p = 0.0286), while no strand bias targeting was observed following generation of the SSB for either the F or R oligonucleotide (p≥0.4857). These results highlight differences between the lesions generated by the wild-type and K223I I-SceI proteins. The lack of strand bias for oligonucleotide targeting upstream or downstream from the break induced by K223I I-SceI support the argument that K223I I-SceI does not generate a DSB but a nick. Furthermore, these data demonstrate that a nick can stimulate gene correction not only at the site of the nick but also at a locus up to 10 kb away.

### An I-SceI DSB, but not an I-SceI SSB, stimulates HR at distant loci in human cells

**Figure 7:**
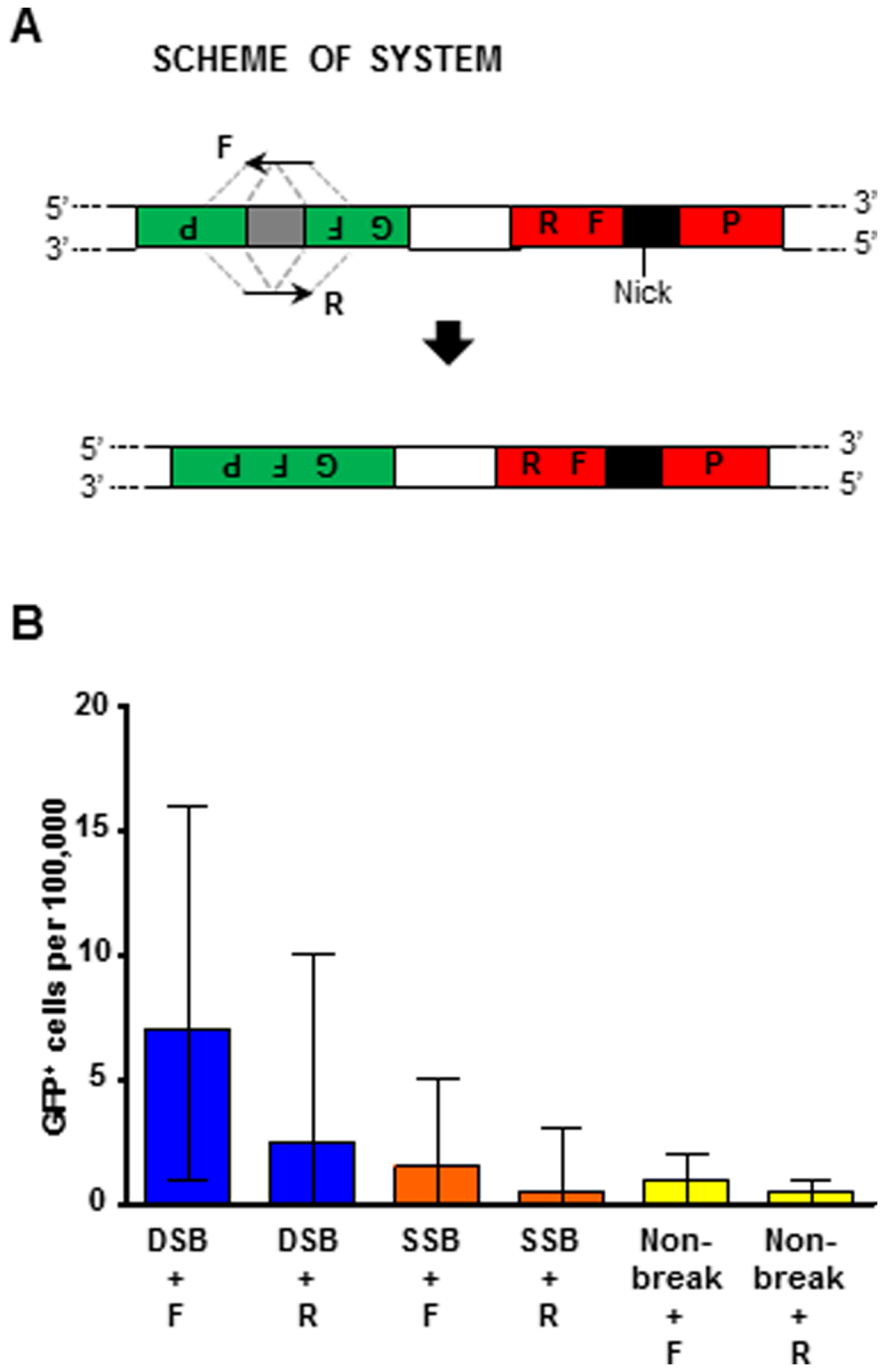
Gene targeting distant from the I-SceI DSB or SSB in human cells. (A) Scheme showing the disrupted GFP plasmid locus 2.3 kb distant from the I-SceI recognition sequence (black box). The position of the SSB is indicated (“Nick”). Dashed gray lines indicate the complementarity between the F oligonucleotide and the antisense strand of the targeted gene, and between the R oligonucleotide and the sense strand of the targeted gene. (B) Frequencies of GFP^+^ cells following expression of wild-type I-SceI (dark blue bars labeled “DSB”), K223I I-SceI (orange bars labeled “SSB”), or D145A I-SceI (yellow bars labeled “Non-break”) using either of the single oligonucleotides to correct the GFP gene distant from the I-SceI break. All data are presented as the median with range (n = 6). For the specific numerical values see Table S3K.

To determine if the I-SceI DSB or SSB could stimulate HR at distant loci in human cells, we utilized plasmid pGRdis containing two disrupted fluorescent protein genes, GFP and RFP (**Figure 7A**). Following transfection with an oligonucleotide designed to restore the sequence of GFP after generation of an I-SceI break in RFP, we examined if a DSB 2.3 kb distant from the targeting locus was able to stimulate HR. We transfected this pGRdis plasmid, a plasmid expressing wild-type, K223I, or D145A I-SceI, and either of the GFP-correcting oligonucleotides into HEK-293 cells and measured green fluorescence by flow cytometry approximately 8 days later. All negative controls produced <1 fluorescent cell per 100,000 cells (<0.25–1). While no increase in fluorescent cells was observed following expression of K223I I-SceI using either oligonucleotide as a repair template (p≥0.2731) or following expression of wild-type I-SceI using R (p = 0.0906), a 9.6-fold increase with the DSB and using the F oligonucleotide was observed at the GFP locus (p = 0.0177) (**Figure 7B**). The results thus show that also in human cells the oligonucleotide that is complementary to the 3′ end of the DSB is the most effective at distant targeting, similarly to what was observed in yeast previously [34], and in this study (**Figure 6A and B**).

## Discussion

The ability to activate the HR machinery through generation of a site-directed break enables efficient genetic modification of genes if a homologous repairing template is provided. While this is most often accomplished following generation of a DSB, the potential for off-site targeting mutations and chromosomal rearrangements that accompany non-homologous repair of the break poses a significant threat for the genomic stability of the treated cells [23]. Though designed double-strand endonucleases, such as ZFNs, TALENs, and CRISPR/Cas proteins, can be optimized for highly specific recognition of a genomic locus, the sequence constraints for such precision may limit the availability of suitable target regions [38], [39]. Conversely, the emergence of designed nickases, including engineered variants of natural meganucleases, such as the nicking variant of I-AniI, as well as zinc finger or CRISPR nickases, as an alternative means of stimulating HR with much less off-site targeting potential holds great promise for gene targeting and correction [25], [27]–[30]. In this study, we characterized a novel variant of wild-type I-SceI, K223I I-SceI, which preferentially cleaves the 18-bp I-SceI recognition site in only one specific position *in vitro* ([31] and **Figure 1A,D**). While wild-type I-SceI has been widely-used for DSB-driven gene targeting, no *in vivo* studies prior to this have explored the capacity of an I-SceI nickase to stimulate HR. We demonstrate here that K223I I-SceI generates a nick which can trigger the HR machinery in yeast and in human cells to stimulate gene correction. Additionally, by directly comparing wild-type and K223I I-SceI in various recombination assays, we demonstrate that the respective DNA lesions generated by these enzymes are activating HR via different mechanisms.

We observed that K223I I-SceI can increase the frequency of HR at genetic loci containing the I-SceI site when expressed in yeast or human cells. While HR frequencies following K223I I-SceI nicking were lower compared to those obtained after cleavage by wild-type I-SceI, expression of K223I I-SceI increased gene correction in yeast cells at the site of the break up to 9-fold. In human cells, up to a 12-fold increase was observed at the site of the K223I I-SceI nick on either of two plasmid loci or on the chromosome (**Figure 5**, panels **B**, **C**, and **D**). These findings demonstrate that the K223I I-SceI nick can stimulate HR *in vivo*, however these results do not rule out the possibility that such HR stimulation is due to a low-level DSB by the K223I I-SceI protein at the recognition site.

Both DSB-induced recombination between direct repeats and gene correction by oligonucleotides at the site of the break in yeast have lower efficiency in the presence of Rad51 [32]–[34]. On the contrary, the K223I nickase stimulated recombination between direct repeats equally well in wild-type and *rad51*Δ cells and promoted gene correction by oligonucleotides in a Rad51-dependent manner (**Figure 2B** and **Figure 3C**). The dependence on Rad51 is not due to a low level of DSB being produced by the K223I I-SceI nickase. Reducing the recombination-stimulating activity of the I-SceI DSB to the level of that observed for K223I I-SceI by using a lower amount of galactose to induce expression of wild-type I-SceI still significantly promoted recombination in the absence of Rad51 (**Figure 3D**). Additionally, while I-SceI DSB gene correction frequencies drastically decreased when cells were transformed following G1 arrest, most likely due to the lack of resection during this stage of the cell cycle [36], there was no difference in the recombination frequency in G1 arrested cells following induction of the I-SceI SSB (**Figure 4**). These results also argue against the observed recombination being due to low levels of DSB formation by the K223I I-SceI protein. Furthermore, in human cells opposite strand biases for repair of the I-SceI SSB or DSB were observed at the GFP locus (**Figure 5**, panels **C** and **D**). Further evidence that the break generated by K223I I-SceI is a nick and not a DSB is seen from the extremely low background of Trp^+^ cells obtained with the no-oligonucleotide control in the presence of K223I I-SceI, in contrast to the high background observed in the presence of wild-type I-SceI even when this stimulates recombination at low levels in low galactose-containing medium (**Figure 3B, C and D** and **Figure 4**). These results suggest that the break caused by wild-type I-SceI can be easily repaired by end joining, but the one generated by K223I I-SceI cannot. Together, these findings indicate differences in the ways wild-type I-SceI and K223I I-SceI stimulate recombination. Thus, although K223I I-SceI infrequently generates a DSB at its specific target site *in vitro* after prolonged incubation with the substrate (**Figure 1D**), this is unlikely to be the stimulus for HR *in vivo*.

We then tested whether a K223I I-SceI could trigger HR at sites distant from the break in yeast and human cells. Highly efficient gene targeting requires generation of a break by a nuclease with sequence recognition next to the targeted genomic locus. However, the sequence specificity of the enzymes utilized to generate the break may limit where a suitable break can be introduced. For example, ZFNs and TALENs require a spacer region between the DNA recognition sequences for each nuclease of the pair [16], [40], and the CRISPR/Cas system requires a protospacer-associated motif (PAM) to be located next to a target locus for directed sequence recognition [19]. Thus, often the only available recognition sequence for these nucleases may be located up to several kb away from the targeted locus to be corrected. The possibility to generate a break to trigger the HR machinery at a distant locus could expand the opportunities for safer gene correction approaches by providing wider windows for mutagenesis. While gene targeting distant from a DSB site has been demonstrated in yeast [34], to our knowledge the potential for correction at a genomic locus distant from an SSB has not been explored until this study. Notably, following generation of the K223I I-SceI SSB in yeast we observed a significant increase in recombinants when the nick was generated either 10 kb upstream or downstream of the genomic locus targeted for correction (**Figure 6**, panels A and B left). Furthermore, while a strand bias for targeting in favor of the oligonucleotide complementary to the 3′ end of the break was observed after generation of the DSB, no bias was observed following SSB generation to stimulate targeting at a locus distant from the break point. In contrast to these findings in yeast, an I-SceI SSB did not stimulate recombination at a distant GFP locus on the pGRdis plasmid in human cells; though it is significant that an I-SceI DSB generated 2.3 kb away was able to trigger HR (**Figure 7B**).

**Figure 8:**
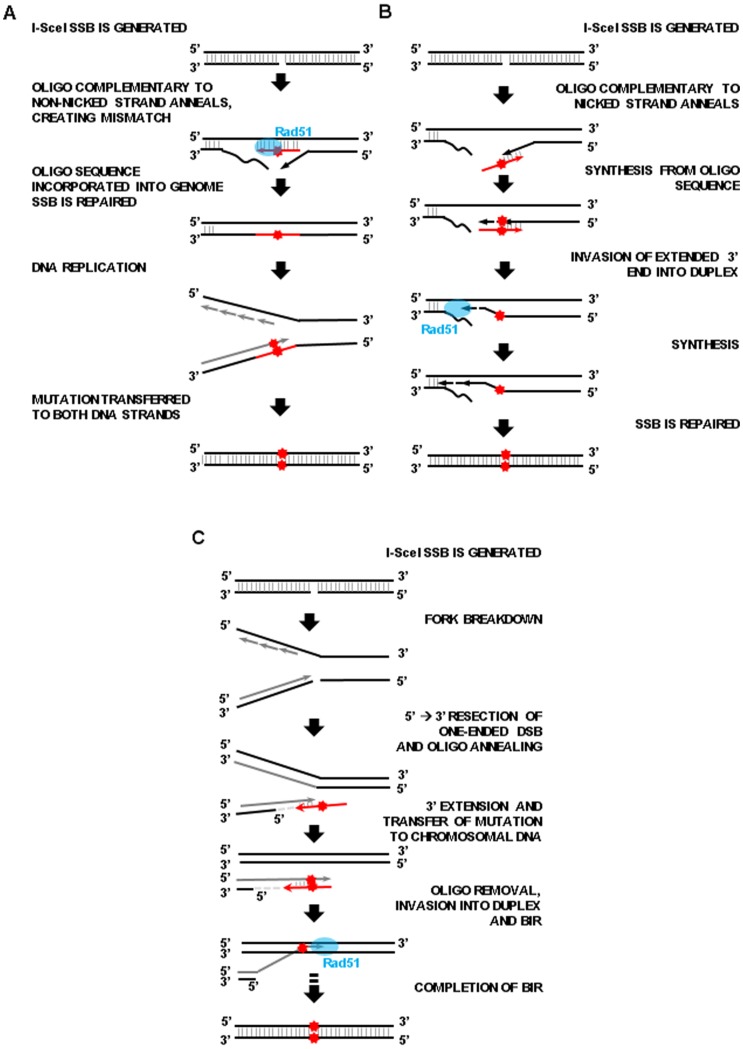
Models for I-SceI K223I SSB-driven HR using single-stranded oligonucleotides. (A) Gene correction using an oligonucleotide complementary to the intact strand. After the SSB is generated, an oligonucleotide (red arrow) carrying the desired nucleotide change (red star) anneals to the complementary strand by invading the nicked duplex with the help of Rad51. The oligonucleotide is incorporated in the duplex and its genetic modification is transferred to the other strand in the subsequent round of DNA replication. (B) Gene correction using an oligonucleotide complementary to the nicked strand. After the SSB is generated, an oligonucleotide sequence (red arrow) with the desired mutation (red star) can serve as template to extend the unwound 3′ broken end. This extended 3′ end may invade the duplex via Rad51 function. The 3′ end is then extended further. The nick is repaired and the mutation fixed by mismatch repair or in the next round of replication. (C) Gene correction following collapse of a replication fork at the nick. After the SSB is generated, it persists until encountered by the replication fork. Following fork breakdown, a one-ended DSB forms. Resection of the 5′ end (indicated by a light gray dashed line) produces a single-strand 3′ end that anneals with the complementary sequence of an oligonucleotide (red arrow) containing a desired mutation (red star) and uses the oligonucleotide as a template for extension. After removal of the oligonucleotide by unwinding or resection, the 3′ end (dark gray) invades the intact duplex via Rad51 and follows the steps (dotted black arrow) of BIR to complete repair. The mutation carried on the 3′end is passed to the other strand of the chromosome by mismatch repair or in the next round of replication.

Our work proves that expression of the K223I I-SceI variant in yeast and human cells stimulates recombination between direct repeats in the yeast genome and by oligonucleotides at chromosomal or plasmid positions in yeast and human cells. We also demonstrate that the molecular path by which the I-SceI nickase stimulates recombination is different from that driven by wild-type I-SceI. We propose two mechanisms for how the I-SceI SSB can stimulate gene targeting by single-stranded oligonucleotides. The first mechanism relies on local unwinding of the nicked strand, in line with what has been proposed for I-AniI nick-induced gene correction by Davis and Maizels [25]. DNA unwinding at the nick exposes chromosomal regions of single-strand DNA and facilitates annealing of a complementary single-stranded oligonucleotide (**Figure 8A and 8B**). In the second mechanism, the nick is converted into a one-ended DSB by a collapsed replication fork (**Figure 8C**), which normally is repaired either by NHEJ or via break-induced replication (BIR) [41]. Resection of the 5′ end of the one-ended DSB can facilitate annealing of a complementary single-stranded oligonucleotide to the 3′ end of the DSB. The 3′ end is then extended using the oligonucleotide as a template, and after unwinding or 5′-end resection of the oligonucleotide, the 3′ end can invade the intact homologous duplex via Rad51 following a BIR mechanism. Both local unwinding next to the nick site and fork collapse at the nick can explain SSB-driven recombination at the break site. Differently, nick-driven recombination at sites distant from the break, up to 10 kb away, most likely follows the model of fork collapse. It is of note that K223I I-SceI nick-driven stimulation of gene targeting at sites distant from the break occurred in the yeast chromosomal system but not in the human plasmid system. Plasmid pGRdis does not have a replication origin that can be activated in human cells and cannot be replicated in HEK-293 cells. It is therefore possible that, in order for a nick to stimulate gene targeting at a distant site, a replication fork must be present and collapse at the nick. If this hypothesis is correct, then a nick induced in a replicating plasmid or on the chromosome could also stimulate HR at a distant locus in human cells. As Rad51 is necessary for HR after generation of a K223I I-SceI SSB in yeast cells, duplex invasion must be a required step (**Figure 8**). Rad51-mediated strand invasion may be facilitated in a nicked DNA (**Figure 8A**), Rad51 may promote the invasion of the extended 3′ end of the nick into the nicked duplex (**Figure 8B**), or Rad51 may stimulate the invasion of the resected one-ended DSB end formed by replication fork breakdown into the intact duplex (**Figure 8C**). Ultimately, our findings show that the K223I I-SceI SSB does stimulate significant recombination at and distant from the site of the break, and provide further evidence that SSB-driven gene targeting is a valuable mechanism through which targeted gene correction can be accomplished.

## Materials and Methods

### Cleavage activity

The double-strand and single-strand DNA cleavage activities of wild-type I-SceI and the K223I and D145A I-SceI variants were analyzed by a kinetics assay using a supercoiled plasmid (pBS-I-SceI (E/H)) containing a single wild-type I-SceI recognition sequence. Reaction mixtures contained I-SceI cleavage buffer (10 mM Tris-HCl (pH 8.8), 1 mM DTT, and 0.1 mg/ml BSA), pBS-I-SceI (E/H) supercoiled plasmid DNA (2.5 nM), and purified wild-type, K223I or D145A I-SceI (100 nM). Reactions were initiated by the addition of MgCl_2_ (15 mM), and the mixtures were incubated for various lengths of time at 30°C before being halted by the addition of EDTA (100 mM). Supercoiled, open-circle and linear DNAs were resolved by gel electrophoresis on a 0.8% agarose gel. Gels were stained with ethidium bromide, and the fluorescence intensities of the DNA bands were determined using a Kodak EDAS 290 imager.

### Yeast plasmids

Plasmid pGSHU [10] contains the GSHU-wild-type-I-SceI cassette (wild-type I-SceI gene under the galactose-inducible *GAL1* promoter, hygromycin resistance gene *hyg*, the counterselectable *KlURA3* marker gene). Plasmids pGSHU-K223I and pGSHU-D145A are derivatives of pGSHU and contain the K223I I-SceI and D145A I-SceI genes, respectively, in place of the wild-type I-SceI gene. The resulting cassettes are referred to as GSHU-wild-type-I-SceI, GSHU-K223I, and GSHU-D145A, respectively.

Plasmid pAG7 [20] is a yeast expression vector containing the *GAL1* promoter, Gene II gene, and selectable *LEU2* marker. Plasmids pAG7-wild-type-I-SceI, pAG7-K223I, and pAG7-D145A, are derivatives of plasmid pAG7 in which the Gene II gene was replaced with the wild-type I-SceI, K223I, or D145A gene, respectively.

Mutagenesis and cloning information are provided as supporting information (Materials and Methods S1).

### Yeast strains

Strains used for the direct repeat assay are derivatives of FRO-830 (*MAT*α *leu2-3,112 his7-2 ura3*Δ *trp1-289 lys2*::DR [GSHU]) [34] and contain a 27-bp insertion comprised of two stop codons and the 18-bp I-SceI site in *lys2* within 90-bp direct repeats flanking either side of the insertion. Strains were generated as previously described [42]. Strains SAS-74 and -75 contain plasmid pAG7-wild-type-I-SceI, SAS-77 and -149 contain plasmid pAG7-K223I, and SAS-142 and -143 contain plasmid pAG7-D145A, respectively. Strains SAS-174 and -175 contain plasmid pAG7-wild-type-I-SceI, SAS-176 and -177 contain plasmid pAG7-K223I, and SAS-178 and -179 contain plasmid pAG7-D145A, respectively, and are *rad51* null mutants in which *RAD51* is replaced with the *kanMX4* cassette.

Strains used for repair at the site of the break are derivatives of FRO-1 (*MAT*α *ade5*-*1 his7*-*2 leu2*-*3*,*112 ura3*-*52 trp5*::*GSHU lys2*::*Alu IR*) [34] and contain one stop codon and the I-SceI site within a 26-bp disruption in *trp5*. Strains were generated as previously described [42]–[45]. For our assays, the site is oriented such that the I-SceI SSB is on the “Crick” chromosomal strand, unless otherwise indicated. The mating type (α) of these strains was switched to mating type **a** to allow for cell cycle arrest with α-factor. Strains SAS-227 and -228 contain plasmid pAG7-wild-typeI-SceI, -229 and -230 contain plasmid pAG7-K223I, and -231 and -232 contain plasmid pAG7-D145A, respectively, and are mating type **a**. Strains SAS-235 and -236 contain plasmid pAG7-wild-type-I-SceI, -237 and -238 contain plasmid pAG7-K223I, and -239 and -240 contain plasmid pAG7-D145A, respectively, and are mating type **a** as well as *rad51* null mutants in which *RAD51* is replaced with the *kanMX4* cassette. To compare if the position of the I-SceI nick in the “Watson” versus “Crick” strand was affecting HR, an additional set of strains was generated such that the SSB can occur on the “Watson” chromosomal strand. These strains are isogenic with those described above, which have the I-SceI site in opposite orientation, such that the I-SceI SSB occurs on the “Crick” strand. Strains SAS-281 and -282 contain plasmid pAG7-wild-type-I-SceI, -283 and -284 contain plasmid pAG7-K223I, and -285 and -286 contain plasmid pAG7-D145A, respectively, and are mating type α. For these strains, a 26-bp disruption sequence containing the 18-bp I-SceI site and two stop codons was inserted into *trp5*. Strains SAS-78 and -79 contain plasmid pAG7-wild-type-I-SceI, -80 and -148 contain plasmid pAG7-K223I, and -116 and -117 contain plasmid pAG7-D145A, respectively, and are mating type α.

Strains used for repair distant from the site of the break are derivatives of FRO-917 (*MAT*α *his3*Δ1 *leu2*Δ0 *lys2*Δ0 *ura3*Δ0 *trp5*::INS31) [34], [46] and contain a nonfunctional *trp5* gene disrupted by a 31-bp frameshift insertion (5′ CCAAATCCTCAGCATAATGATTAGGTATGCA) [34]. Diploid strains SAS-150 and -151, -162 and -163, and -166 and -167 derive from crossings of FRO-879 (isogenic to FRO-917), in which *TRP5* is replaced by *LEU2*, with FRO-872, FRO-138, or FRO-140, respectively, and contain the GSHU-wild-type-I-SceI, GSHU-K223I, and GSHU-D145A cassettes, respectively, positioned approximately 10 kb upstream of the 31-bp disruption in *trp5*, and containing the I-SceI site oriented such that the SSB is made on the “Crick” chromosomal strand (**Table S1**). Diploid strains SAS-215 and -217, -207 and -209, and -211 and -213 contain the GSHU-wild-type-I-SceI, GSHU-K223I, and GSHU-D145A cassettes, respectively, positioned approximately 10 kb upstream of the 31-bp disruption in *trp5*, and containing the I-SceI site oriented such that the SSB is made on the “Watson” chromosomal strand. Diploid strains SAS-152 and -153, -154 and -156, and -158 and -160 contain the GSHU-wild-type-I-SceI, GSHU-K223I, and GSHU-D145A cassettes, respectively, positioned approximately 10 kb downstream of the 31-bp disruption in *trp5*, and containing the I-SceI site oriented such that the SSB is made on the “Watson” chromosomal strand. Diploid strains SAS-272 and -274, -219 and -221, and -251 and -253 contain the GSHU-wild-type-I-SceI, GSHU-K223I, and GSHU-D145A cassettes, respectively, positioned approximately 10 kb downstream of the 31-bp disruption in *trp5*, and containing the I-SceI site oriented such that the SSB is made on the “Crick” chromosomal strand.

The GSHU-wild-type-I-SceI cassette used in this study was PCR-amplified from plasmid pGSHU using primers described previously [10]. The GSHU-K223I and GSHU-D145A cassettes were amplified from plasmids pGSHU-K223I and pGSHU-D145A, respectively, using these same primers. The integration of the cassette into the yeast genome was carried out as previously described [10], [42].

Genetic methods and standard media were described previously [42], [47]. Strain genotypes are presented in Table S1. Additional details on strain construction are provided in **Materials and Methods S1**.

### Direct repeat repair

For the assay to correct the non-functional *lys2* locus, cells were grown overnight in 5 ml yeast extract-peptone-lactic acid (YPLac) liquid medium at 30°C with shaking. Appropriate dilutions of the overnight cultures were made, and cells were plated directly to synthetic complete medium lacking lysine (SC-Lys) containing 2% galactose to express *GAL1*-I-SceI and induce the break. Cells were also plated to synthetic complete (SC) medium to assess cell viability.

### Break induction and targeting using oligonucleotides

Break induction and targeting with oligonucleotides were performed as previously described [42], [46], [47]. Briefly, for targeting at the *trp5* locus, 50 ml of an overnight YPLac liquid culture was inoculated with galactose (2% final concentration, unless otherwise noted) and incubated with vigorous shaking at 30°C for 4 h (7 h for experiments involving modification distant from the break) to express *GAL1*-I-SceI and induce the break. After incubation in galactose, cells were prepared for transformation. Transformation with lithium acetate was done using ∼5×10^7^ cells/ml with 1 nmol of total oligonucleotide DNA [42]. Oligonucleotides were not annealed prior to co-transformation. Sequences of oligonucleotides used to repair the *trp5* gene are listed in **Table S2**. Cells from each oligonucleotide transformation were diluted appropriately and spread directly to synthetic complete medium lacking tryptophan (SC-Trp) to select for oligonucleotide-mediated targeted gene correction of *trp5* or to SC to assess cell viability.

For the assay in which wild-type-I-SceI stimulation of gene targeting was diminished, a final concentration of 0.02% galactose was inoculated into the overnight culture prior to shaking at 30°C for 4 h.

### Cell cycle arrest

Mating type **a** cells were arrested in G1 as follows: α-factor (US Biological, Marblehead, MA) was transferred into a 50 ml overnight culture of YPLac for a final concentration of 0.4 µg/ml. Cultures were incubated with vigorous shaking at 30°C for 2 h. Cells were sonicated and the percentage of G1 arrested cells was determined by counting 200 cells per culture using an hemocytometer. After 2 h, >80% of cells were in G1.

### Human plasmids

Plasmid pSce (a kind gift from M. Porteus, Stanford University) contains the I-*Sce*I endonuclease gene regulated by a CMV/CBA hybrid promoter [12]. Plasmid pSce-K223I, which contains the K223I gene, and plasmid pSce-D145A, which contains the D145A gene, are derived from plasmid pSce and also contain the CMV/CBA hybrid promoter.

Plasmid pGRdis contains two nonfunctional reporter genes, eGFP and DsRed2, both under the CMV promoter oriented opposite from each other and separated by a 300-bp spacer. Within eGFP there is a 148-bp insertion containing two stop codons, a unique XhoI site, and the recognition sequence for the HO endonuclease from *S. cerevisiae*. Within DsRed2 there is a 37-bp insertion containing two stop codons, a unique XbaI site, and the recognition sequence for I-SceI. Additionally, the recognition sequences of HO and I-SceI are located approximately 2.3 kb from each other. This plasmid was used for experiments assaying repair at the site of the break in DsRed2 as well as for the human distant repair assays.

Plasmid pA658 (a kind gift from M. Porteus, Stanford University) contains a GFP gene disrupted by a 35-bp insert containing a single stop codon, and the recognition site for the I-SceI endonuclease [12]. This plasmid was used for experiments assaying repair at the site of the break in GFP.

Mutagenesis and cloning information are provided in **Materials and Methods S1**.

### Human cell lines and culture

HEK-293 cells were grown in Dulbecco's modified Eagle's medium, DMEM (Mediatech, Inc., Manassas, VA), supplemented with 10% heat-inactivated fetal bovine serum (Gemini, Bio-Products, West Sacramento, CA) and 1× penicillin/streptomycin (Lonza, Walkersville, MD). Cells were grown at 37°C in a 5% CO_2_ humidified incubator. Cell line 293/A658 (kindly provided by M. Porteus, Stanford University) is a HEK-293 derivative cell line containing a randomly integrated copy of the sequence of plasmid pA658 [12]. Cells were transfected using polyethylenimine (PEI, Polysciences, Warrington, PA) transfection reagent in 24-well plates at a density of ∼50,000 cells per well as previously described [37]. In all transfection experiments in HEK-293 cells, plasmid DNA was used in the amount of 0.5 µg and oligonucleotide DNA was 1.5 µg, while 1 µg each of oligonucleotide and plasmid DNA was used for transfection experiments in 293/A658 cells. In these experiments, the oligonucleotides and the plasmid were diluted in DMEM without supplements, vortexed in the presence of PEI, and then added to the wells 20 min later. Green or red fluorescent cells were visualized by fluorescent microscopy using a Zeiss Observer A1 microscope and an AxioCamMRm camera (Zeiss, Thornwood, NY). Frequencies of GFP^+^ cells were obtained 5–8 days following transfection by flow cytometric analysis using the BD LSR II Flow Cytometer (BD Biosciences, Sparks, MD). Frequencies of RFP^+^ cells were obtained 5–8 days following transfection by flow cytometric analysis using the BD FACS Aria Cell Sorter (BD Biosciences). 100,000 cells were counted for each sample. Sequences of oligonucleotides used to repair the GFP or DsRed2 genes are listed in **Table S2**.

### Data presentation and statistics

The statistical methodology used to analyze the experimental results is based on non-parametric analysis [48]. For all histograms, data are plotted as median values with the range shown. Statistical significance was determined by using the Mann-Whitney U test [48]. Graphs were created using GraphPad Prism 5 (GraphPad Software, Inc.). Data presenting the values that are plotted on the all the graphs of this study are included in **Table S3**.

## Supporting Information

Figure S1 **Result of BamHI restriction digestion following colony PCR.** The *TRP5* locus was PCR amplified from Trp^+^ colonies formed after transformation of cells expressing wild-type (SAS-78) or K223I (SAS-148) I-SceI without or with the TRP5.80F and TRP5.80R complementary pair of oligonucleotides, which introduce a silent mutation generating a BamHI site upon recombination into the trp5 locus. The PCR product (∼880 bp) was then digested with BamHI. Lanes 1 and 17, 2-log DNA ladder (NEB). Lane 2, undigested PCR product. Lane 3, uncut PCR product deriving from Trp^−^ SAS-78. Lanes 4 and 5, uncut PCR product deriving from Trp^+^ transformant clones of SAS-78 expressing wild-type I-SceI and transformed with no oligonucleotides; lanes 6–10, cut PCR product deriving from Trp^+^ transformant clones of SAS-78 expressing wild-type I-SceI and transformed with TRP5.80F and TRP5.80R. Lane 11, uncut PCR product deriving from Trp^−^ SAS-148. Lanes 12–16, cut PCR product deriving from Trp^+^ transformant clones of SAS-148 expressing K223I I-SceI and transformed with TRP5.80F and TRP5.80R. The PCR products that are digested and cut by BamHI into 546-bp and 317-bp bands still retain uncut product; this is due to the fact the PCR product is directly digested without being first purified.
(DOCX)

Materials and Methods S1 **The section provides details on the construction of yeast plasmids, yeast strains and human plasmids used in this study.**
(DOCX)

Table S1 **Strains used for yeast studies.** ^a^ *lys2*::DR[GSHU] contains the GSHU cassette (wild-type- I-SceI gene and I-SceI site, *hyg* marker conferring resistance to hygromycin B, and *URA3* gene) within 90-bp direct repeats inside the coding sequence of the *LYS2* gene. ^b^ *lys2*::DR I-SceI site contains only the 18-bp I-SceI site within 90-bp direct repeats inside the coding sequence of the *LYS2* gene. ^c^Plasmids expressing wild-type, K223I, or D145A I-SceI (pAG7-wild-type-I-SceI, pAG7-K223I, or pAG7-D145A, respectively) were transformed separately into yeast cells to generate different strains. ^d^Single gene deletions of *RAD51* were generated through targeted replacement with the *kanMX4* cassette conferring resistance to G418. ^e^Strain SAS-59 and its derivatives are mating type “α” and contain the I-SceI site inside the coding sequence of the *TRP5* gene oriented such that an I-SceI SSB is generated on the “Crick” strand. ^f^Strain SAS-193 and its derivatives are mating type “**a**” and contain the I-SceI site inside the coding sequence of the *TRP5* gene oriented such that an I-SceI SSB is generated on the “Crick” strand. ^g^Strain SAS-278 and its derivatives are mating type “α” and contain the I-SceI site inside the coding sequence of the *TRP5* gene oriented such that an I-SceI SSB is generated on the “Watson” strand. ^h^Strain FRO-917 and its derivatives contain a 31-bp insert inside the coding sequence of the *TRP5* gene. ^i^The GSHU-wild-type-I-SceI, GSHU-K223I, or GSHU-D145A cassettes were inserted into the genome approximately 10 kb upstream of the *trp5* locus along with the I-SceI site such that an I-SceI SSB is generated on the “Watson” strand. ^j^The GSHU-wild-type-I-SceI, GSHU-K223I, or GSHU-D145A cassettes were inserted into the genome approximately 10 kb upstream of the *trp5* locus along with the I-SceI site such that an I-SceI SSB is generated on the “Crick” strand. ^k^The GSHU-wild-type-I-SceI, GSHU-K223I, or GSHU-D145A cassettes were inserted into the genome approximately 10 kb downstream of the *trp5* locus along with the I-SceI site such that an I-SceI SSB is generated on the “Watson” strand. ^l^The GSHU-wild-type-I-SceI, GSHU-K223I, or GSHU-D145A cassettes were inserted into the genome approximately 10 kb downstream of the *trp5* locus along with the I-SceI site such that an I-SceI SSB is generated on the “Crick” strand.
(DOCX)

Table S2 **Oligos used for repair assays.** The sequence of the oligos used for repair of the disrupted *trp5* (yeast), GFP (human cells), or DsRed2 (human cells) loci are listed from the 5′ ends of each 80-base sequence.
(DOCX)

Table S3 **Supplementary data for graphs.** Data from Figure 2B. Data are presented as the median with the range in parentheses; n≥11. Results were statistically analyzed using the Mann-Whitney U test. ^a^Strains used: *RAD51* (SAS-74 and SAS-75) and *rad51*Δ (SAS-174 and SAS-175). ^b^Strains used: *RAD51* (SAS-77 and SAS-149) and *rad51*Δ (SAS-176 and SAS-177). ^c^Strains used: *RAD51* (SAS-142 and SAS-143) and *rad51*Δ (SAS-178 and SAS-179). ** p≤0.01, **** p≤0.0001, NS  =  not significant, n/a (not applicable).
(DOCX)
