# Supplementary material for: To Nick or Not to Nick: Comparison of I-SceI Single- and Double-Strand Break-Induced Recombination in Yeast and Human Cells
Source: PLoS One. 2014 Feb 18;9(2):e88840. doi: 10.1371/journal.pone.0088840 (PMC3928301; doi:10.1371/journal.pone.0088840)
Supplement: Figure S1 — Result of BamHI restriction digestion following colony PCR. The TRP5 locus was PCR amplified from Trp+ colonies formed after transformation of cells expressing wild-type (SAS-78) or K223I (SAS-148) I-SceI without or with the TRP5.80F and TRP5.80R complementary pair of oligonucleotides, which introduce a silent mutation generating a BamHI site upon recombination into the trp5 locus. The PCR product (∼880 bp) was then digested with BamHI. Lanes 1 and 17, 2-log DNA ladder (NEB). Lane 2, undigested PCR product. Lane 3, uncut PCR product deriving from Trp− SAS-78. Lanes 4 and 5, uncut PCR product deriving from Trp+ transformant clones of SAS-78 expressing wild-type I-SceI and transformed with no oligonucleotides; lanes 6–10, cut PCR product deriving from Trp+ transformant clones of SAS-78 expressing wild-type I-SceI and transformed with TRP5.80F and TRP5.80R. Lane 11, uncut PCR product deriving from Trp− SAS-148. Lanes 12–16, cut PCR product deriving from Trp+ transformant clones of SAS-148 expressing K223I I-SceI and transformed with TRP5.80F and TRP5.80R. The PCR products that are digested and cut by BamHI into 546-bp and 317-bp bands still retain uncut product; this is due to the fact the PCR product is directly digested without being first purified. (DOCX) [file pone.0088840.s001.docx]

**Supplementary Figure S1.** **Restriction digestion of PCR products by BamHI.**


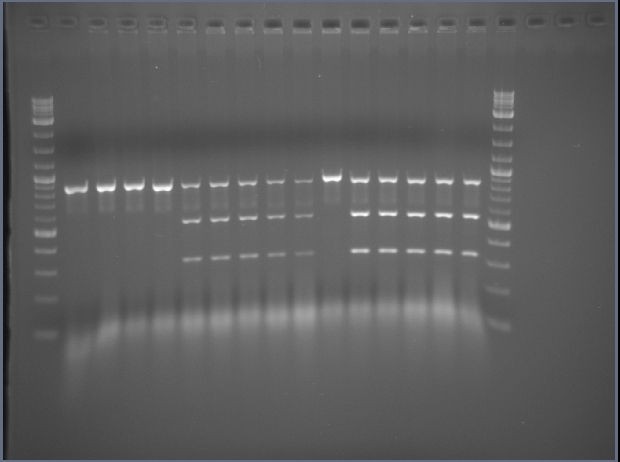


**317 bp**

**546 bp**

**880 bp**

**17**

**16**

**15**

**14**

**13**

**12**

**11**

**10**

**7**

**9**

**8**

**5**

**4**

**6**

**3**

**2**

**1**

**Figure S1**. Result of BamHI restriction digestion following colony PCR. The *TRP5* locus was PCR amplified from Trp^+^ colonies formed after transformation of cells expressing wild-type (SAS-78) or K223I (SAS-148) I-SceI without or with the TRP5.80F and TRP5.80R complementary pair of oligonucleotides, which introduce a silent mutation generating a BamHI site upon recombination into the trp5 locus. The PCR product (~880 bp) was then digested with BamHI. Lanes 1 and 17, 2-log DNA ladder (NEB). Lane 2, undigested PCR product. Lane 3, uncut PCR product deriving from Trp^-^ SAS-78. Lanes 4 and 5, uncut PCR product deriving from Trp^+^ transformant clones of SAS-78 expressing wild-type I-SceI and transformed with no oligonucleotides; lanes 6-10, cut PCR product deriving from Trp^+^ transformant clones of SAS-78 expressing wild-type I-SceI and transformed with TRP5.80F and TRP5.80R. Lane 11, uncut PCR product deriving from Trp^-^ SAS-148. Lanes 12-16, cut PCR product deriving from Trp^+^ transformant clones of SAS-148 expressing K223I I-SceI and transformed with TRP5.80F and TRP5.80R. The PCR products that are digested and cut by BamHI into 546-bp and 317-bp bands still retain uncut product; this is due to the fact the PCR product is directly digested without being first purified.
