# Supplementary material for: To Nick or Not to Nick: Comparison of I-SceI Single- and Double-Strand Break-Induced Recombination in Yeast and Human Cells
Source: PLoS One. 2014 Feb 18;9(2):e88840. doi: 10.1371/journal.pone.0088840 (PMC3928301; doi:10.1371/journal.pone.0088840)
Supplement: Materials and Methods S1 — The section provides details on the construction of yeast plasmids, yeast strains and human plasmids used in this study. (DOCX) [file pone.0088840.s002.docx]

**Materials and Methods S1**

**Yeast plasmid construction**

Plasmid pGSHU, described previously [34], contains the GSHU-wild-type I-SceI cassette (I-SceI gene under the *GAL1* promoter, hygromycin resistance gene *hyg*, the counterselectable *KlURA3* marker gene) and was used as a template to construct plasmids pGSHU-K223I and pGSHU-D145A, containing the K223I I-SceI and D145A I-SceI genes, respectively. Site-directed mutagenesis was conducted using the QuikChange II Site-Directed Mutagenesis Kit (Agilent Technologies, Inc., Santa Clara, CA). Point mutations were generated by primers I-SceI K223I.F (5’-GCAGATGATGTACATTCTGCCGAACACTAT) and I-SceI K223I.R (5’-ATAGTGTTCGGCAGAATGTACATCATCTGC) to make plasmid pGSHU-K223I (AAA🡪ATT at I-SceI residue 233) or primers I-SceI D145A.F (5’-Atactggttcatggctgatggtggtaaat) and I-SceI D145A.R (5’-ATTTACCACCATC

AGCCATGAACCAGTAT) to make plasmid pGSHU-D145A (GAT🡪GCT at I-SceI residue 145) containing the wild-type I-SceI variants described by Niu *et al*. [31]. Plasmids were rescued in *Escherichia coli* and confirmed by sequence analysis.

Plasmids pAG7-wild-typeI-SceI, pAG7-K223I, and pAG7-D145A were constructed as follows. Plasmid pAG7 [20] was digested with BamHI to excise the ~1.3 kb fragment containing the coding sequence of Gene *II*. A 750-bp fragment containing the coding sequence of the I-SceI gene from plasmids pGSHU, pGSHU-K223I, or pGSHU-D145A was amplified through PCR using primers pAG7 clone I-SceI.F (5’-ACCCCGGATCCCGGGGGTACATATGCATATGAA

AAACATCAA) and pAG7 clone I-SceI.R (5’-CTAGAGGATCCCCGGGTACCGAGCTCGAA

TTCCCTTATTTCAGGAAAGTTTCGG) and cloned into the BamHI site of the digested pAG7 plasmid resulting in plasmids pAG7-wild-typeI-SceI, pAG7-K223I, or pAG7-D145A, respectively. Plasmids were rescued in *E. coli* and confirmed by sequence analysis.

**Yeast strain construction**

The strain FRO-830 (*MAT*α *leu2-3,112 his7-2 ura3Δ trp1-289 lys2*::DR) contains the GSHU-wild-typeI-SceI cassette and the 18-bp I-SceI site in *lys2* within 90-bp direct repeats flanking either side of the cassette. Strains used for the direct repeat experiments are derivatives of FRO-830 and were generated as previously described [42-47]. Briefly, the entire GSHU-wild-typeI-SceI cassette was replaced with the full I-SceI site using oligos LYS2 I-SceIsite.F (5’- CACTGGGTTTATCCATATGCCAAATTGAGCTAATAAattaccctgttatccctaAA

GAGAAGTGGATGGATTTGGCAAACACAGTT) and LYS2 I-SceIsite.R (5’-AACTGTGTTTGCCAAATCCATCCACTTCTCTTTAGGGATAACAGGGTAATTTATTAGTCAATTTGGCATATGGATAAACCCAGTG) to produce strain SAS-50. Plasmids containing the galactose-inducible *GAL1* promoter and variants of the I-SceI gene were then transformed into this strain to yield the following strains: SAS-74 and -75, which contain pAG7-wild-typeI-SceI; SAS-77 and -149, which contain pAG7-K223I; and SAS-142 and -143, which contain pAG7-D145A. Additionally, in strain SAS-50 the entire coding region of *RAD51* was replaced with the *kanMX4* cassette conferring antibiotic resistance to G418 for the *rad51*Δ mutants. For the selection of G418-resistant colonies, cells were grown on yeast extract-peptone-dextrose (YPD) medium containing 200 μg/ml of G418 (Mediatech, Inc., Manassas, VA). Plasmids were transformed into the resulting strain to yield the following *rad51*Δ mutant strains: SAS-174 and -175, which contain pAG7-wild-typeI-*Sce*I; SAS-176 and -177, which contain pAG7-K223I; and SAS-178 and -179, which contain pAG7-D145A. Leu^+^ transformants were selected for on synthetic complete medium lacking leucine (SC-Leu) and subsequently maintained on this medium to retain the plasmid.

The strain FRO-1 (*MAT*α *ade5*-*1 his7*-*2 leu2*-*3*,*112 ura3*-*52 trp5::GSHU lys2::Alu IR*) contains the GSHU-wild-typeI-SceI cassette and the I-SceI site within *trp5*. Strains used for repair at the site of the break are derivatives of FRO-1 and were generated as previously described [42-47]. Briefly, the entire GSHU-wild-typeI-SceI cassette was replaced with the full I-SceI site oriented such that a nick created by the I-SceI nickase is made on the “Crick” chromosomal strand, using oligos TRP5 I-SceI site. F (5’-gaagtcttcccagaatgtggga

tcgattaccctgttatccctatgatgaAAgcgacagcttcatcaaaaccctttt) and TRP5 I-SceI site.R (5’-aaaagggttttgatgaagctgtcgcTTtcatcaTAGGGATAAC

AGGGTAATcgatcccacattctgggaagactt) resulting in strain SAS-59, or on the “Watson” chromosomal strand, using oligos TRP5 I-SceI site opp.F (5’ –aaaagggttttgatgaagctgtcgcatgatgaattaccctgttatccctaacgatcc

cacattctgggaagacttc) and TRP5 I-SceI site opp.R (5’ –GAAGTCTTCCCAGAATGT

GGGATCGTTAGGGATAACAGGGTAATTCATCATGCGACAGCTTCATCAAAACCCTTTT) resulting in strain SAS-278. Plasmids containing the galactose-inducible *GAL1* promoter and variants of the I-SceI gene were then transformed into these strains to yield the following strains. Strains in which the I-SceI nick is generated on the “Crick” strand: SAS-78 and -79, which contain pAG7-wild-typeI-SceI; SAS-80 and -148, which contain pAG7-K223I; and SAS-116 and -117, which contain pAG7-D145A. Strains in which the I-SceI nick is generated on the “Watson” strand: SAS-281 and -282, which contain pAG7-wild-typeI-SceI; SAS-283 and -284, which contain pAG7-K223I; and SAS-285 and -286, which contain pAG7-D145A. For strains containing the nicking position on the “Crick” strand the mating type was then switched as follows. The coding region of the *BAR1* gene was replaced with the *URA3* gene, and Ura^+^ transformants were selected on synthetic complete medium lacking uracil (SC-Ura), yielding strain SAS-182. Plasmid pHO-LEU2, which contains the mating type switching endonuclease HO regulated by the inducible *GAL1* promoter and selectable *LEU2* marker, was then transformed into this strain, and transformants were selected for on SC-Leu. Cells were then grown in yeast extract-peptone-galactose (YPGal) liquid medium for one hour at 30°C to allow for expression of the HO protein and dilutions were plated to YPD solid medium. The resulting strain, SAS-193, and its derivative, SAS-205, in which the entire coding region of *RAD51* was replaced with the *kanMX4* cassette, were transformed with plasmids containing the galactose-inducible *GAL1* promoter and variants of the I-SceI gene. *RAD51* strains: SAS-227 and -228, which contain pAG7-wild-typeI-*Sce*I; SAS-229 and -230, which contain pAG7-K223I; and SAS-231 and -232, which contain pAG7-D145A. *rad51*Δ strains: SAS-235 and -236, which contain pAG7-wild-typeI-*Sce*I; SAS-237 and -238, which contain pAG7-K223I; and SAS-239 and -240, which contain pAG7-D145A. All strains were grown on synthetic complete medium lacking leucine (SC-Leu) following transformation to maintain the plasmid.

The strain FRO-917 is a derivative of BY4742 (*MAT*α *his3Δ1 leu2Δ0 lys2Δ0 ura3Δ0 trp5::*INS31) and contains a non-functional *trp5* gene disrupted by a 31-bp insert [34]. Strains FRO-872 [34], SAS-138, and -140 contain a GSHU-wild-type I-SceI, GSHU-K223I, or GSHU-D145A cassette (and I-SceI site on the “Crick” strand), respectively, which was amplified as a PCR product with primers 10KbUP.IS (5-AGATAATTTACCCTTGCTTTAAGCTGCGTATAT

CAAGTGCATTTGCTGTCTAGGGATAACAGGGTAATTTCGTACGCTGCAGGTCGAC) and 10KbUP.II (5’-TCGTTCGTTATCCGAAGCTGGCCAATTGATACAATTAATTGACATC

AGCATTGGATGGACGCAAAGAAGT) and cloned approximately 10 kb upstream of the 31-bp insert in *trp5*. Strains SAS-199 and -200, -195 and -196, and -197 and -198 contain a GSHU-wild-type I-SceI, GSHU I-SceI-K223I, or GSHU I-SceI-D145A cassette (and I-SceI site on the “Watson” strand), respectively, which was amplified as a PCR product with primers 10KbUP.opp.IS (5’ –AGATAATTTACCCTTGCTTTAAGCTGCGTATATCAAGTGCATTTG

CTGTCATTACCCTGTTATCCCTATTCGTACGCTGCAGGTCGAC) and 10KbUP.II and cloned approximately 10 kb upstream from the 31-bp insert in *trp5*. The diploid strains SAS-150 and -151, -162 and -163, -166 and -167, -215 and -217, -207 and -209, and -211 and -213 were generated by mating FRO-872 with FRO-879, SAS-138 and FRO-879,SAS-140 with FRO-879, SAS-199 or -200 with FRO-879, SAS-195 or -196 with FRO-879, and SAS-197 or -198 with FRO-879 respectively. FRO-879 is BY4741 (*MAT***a** *his3Δ1 leu2Δ0 met15Δ0 ura3Δ0*), where *TRP5* has been replaced by *LEU2* [34]. Strains FRO-876 [34], SAS-134 and -135, and -136 and -137 contain a GSHU-wild-type I-SceI, GSHU-K223I, or GSHU-D145A cassette (and I-SceI site on the “Watson” strand), respectively, which was amplified as a PCR product with primers 10KbDW.IS (5’-CCGAGGTATTGTTGTATATACCACTACTCTGTGATTTTTTTTCACTCT

TGTAGGGATAACAGGGTAATTTCGTACGCTGCAGGTCGAC) and 10KbDW.II (5’-TTTGTCATTAGACACTTACCAGTTGATGTTTTCACTTTTTCTTTCCTTCCTTGGATGGACGCAAAGAAGT) and cloned approximately 10 kb downstream of the 31-bp insert in *trp5.* Strains SAS-269 and -270, SAS-201 and -202, and -245 and -246 contain a GSHU-wild-type I-SceI, GSHU-K223I, or GSHU-D145A cassette (and I-SceI site on the “Crick” strand), respectively, which was amplified as a PCR product with primers 10KbDW.opp.IS (5’ –CCGAGGTATTGTTGTATATACCACTACTCTGTGATTTTTTTTCACTCTTGATTACCCTGTTATCCCTATTCGTACGCTGCAGGTCGAC) and 10KbDW.II and cloned approximately 10 kb downstream from the 31-bp insert in *trp5.* The diploid strains SAS-152 and -153, -154 and -156, -158 and -160, -272 and -274, -219 and -221, and -251 and -253 were generated by mating FRO-876 with FRO-879, SAS-134 or -135 and FRO-879,SAS-136 or -137 with FRO-879, SAS-269 or -270 with FRO-879, SAS-201 or -202 with FRO-879, and SAS-245 or -246 with FRO-879, respectively.

**Human plasmid construction**

Plasmids pET15b I-SceI and pET15b I-SceI-K223I contain the wild-type I-SceI and K223I I-SceI genes described previously [31]. These plasmids were digested with NsiI and BamHI to produce a ~740-bp fragment containing the I-SceI gene of interest which was cloned into pFLAG-CMV-6c (Sigma Aldrich, St. Louis, MO) between the BamHI and PstI sites. This yielded plasmids pSD1 (wild-type I-SceI) and pSS1 (K223I I-SceI). Plasmid pSD1 was then used as a template for site-directed mutagenesis using primers I-SceI D145A.F and I-SceI D145A.R to generate plasmid pSBO1, which contains the D145A mutation yielding the D145A gene. Plasmid pSce (a kind gift from M. Porteus, Stanford University) contains the wild-type I-SceI endonuclease gene regulated by a CMV/CBA hybrid promoter [12] and was used as a template to construct plasmids pSce-K223I and pSce-D145A. First, fragments of the K223I and D145A genes from the internal HindIII site to the stop codon were amplified with primers p67clone HindIII.F (5’–tgtagtacagtcatacgcgcatacAAGCTTtcaacaaactggc

taacctg) and p67 Clone SceISalI.R (5’–atcgtagtcgtatGTCGACGAATTCttattat

ttcaggaaagtttcggag) and blunt-end cloned into the pCR-Blunt II-TOPO vector (Invitrogen, Carlsbad, CA). Next, these plasmids were digested with HindIII and SalI to produce 400-bp fragments. Plasmid pSce was also digested with these two enzymes, and the 400-bp fragments were cloned into the vector. All plasmids were rescued in *E. coli* and confirmed by sequence analysis.

Plasmid pGRdis contains two nonfunctional reporter genes, eGFP and DsRed2, both under the CMV promoter oriented opposite from each other and separated by a 300-bp spacer. It was constructed as follows. A fragment, containing the entire coding sequence of eGFP and amplified as a ~720-bp amplicon through PCR using primers GFP clone pSILENCE.F (5’–AAAAAGGATCCATGGTGAGCAAGGGCGAGGA) and GFP clone pSILENCE.R (5 –AAAAAAAGCTTTTACTTGTACAGCTCGTCCA), was digested with BamHI and HindIII and cloned into plasmid pSilencer (Invitrogen) between the BamHI and HindIII sites to produce plasmid pEGFP17. PrimersEGFP disXho.F (5’–ACGACGGCAACTACAAGACCTGATAAG

GCTCGAGCGCGCCGAGGTGAAGTTCGA) and EGFP disXho.R (5’–TCGAACTTCACCT

CGGCGCGCTCGAGCCTTATCAGGTCTTGTAGTTGCCGTCGT) were used to disrupt eGFP between residues 109 and 110 to insert two stop codons, an XhoI site, and 2 frameshift bases through *in vitro* mutagenesis, producing plasmid pdisEGFP3. Next, using primers 300 bp BamHIbuff.F (5’–TTTCTTCTCAGGATCCGGGTCCATACATTTGCCTTT) and 300 Bam buff.R (5’–AAGCTCGATCGGATCCATCAATAATCCCCTTGGTTC) a 300-bp amplicon with BamHI tails generated through PCR of part of the *URA3* gene from *Kluyveromyces lactis* was inserted into plasmid pdisEGFP3 at the BglII site through compatible cloning, generating plasmid pdisEGFP300. Separately, plasmid pDsRed2-N1 (Clontech, Mountain View, CA) was modified to generate the disrupted DsRed2 marker. Part of the MCS was removed through compatible cloning following double digestion with BamHI and BglII. Next, DsRed2 was linearized at SbfI, and using primers ISceIstop_SbfI.F (5’-ACAGTGCTAAGTCCTGCAGG

ACTAATAGTCTAGAATTACCCTGTTATCCCTAAGCTTCCTGCAGGTAGGAACTCAAT) and ISceIstop_SbfI.R (5’-ATTGAGTTCCTACCTGCAGGAAGCTTAGGGATAACAGGGTA

ATTCTAGACTATTAGTCCTGCAGGACTTAGCACTGT) a fragment containing two stop codons and the 18-bp I-SceI site was cloned in. Next, site-directed mutagenesis was used to remove one of the flanking SbfI sites with primers DeltaSbfI.F (5’ –AGAATTACCCTGTTATC

CCTAAGCTTACGGCTGCTTCATCTACAAGGTGAAG) and DeltaSbfI.R(5’–CTTCACCTT

GTAGATGAAGCAGCCGTAAGCTTAGGGATAACAGGGTAATTCT). The construct was amplified through PCR with primers DsRed2-EGFPclone.F (5’–ACAGTGCTAAGTGGATCCA

CCGTATTACCGCCATGCAT) and DsRed2-EGFPclone.R (5’–ATTGAGTTCCTAGAATTCA

TCTCGGTCTATTCTTTTGA) and blunt-end cloned into the pCR-Blunt II-TOPO vector. A ~1.8 kb fragment containing the CMV promoter and disrupted DsRed2 gene was excised by digestion with EcoRI and cloned into plasmid pdisEGFP300, linearized by EcoRI, to produce plasmid pdisEGFP300-disDsRed2. Finally, a ~150-bp fragment, containing the HO endonuclease recognition site and amplified through PCR using primers HO site XhoI.F (5’-ATCAAGCTAGCTCGAGGGTACGGGGATCTAAATAAATTCGTTTTCA) and HO site SalI.R (5’–CGATATCGAAGTCGACTACAACCACTCTACAAAACCAAAACCAGGG), was digested with XhoI and SalI. Plasmid pdisEGFP300-disDsRed2 was linearized by XhoI, and the fragment was cloned into this site, leaving a unique XhoI site on the plasmid. All plasmids were rescued in *E. coli* and confirmed by sequence analysis.
