# Supplementary material for: To Nick or Not to Nick: Comparison of I-SceI Single- and Double-Strand Break-Induced Recombination in Yeast and Human Cells
Source: PLoS One. 2014 Feb 18;9(2):e88840. doi: 10.1371/journal.pone.0088840 (PMC3928301; doi:10.1371/journal.pone.0088840)
Supplement: Table S1 — Strains used for yeast studies. a lys2::DR[GSHU] contains the GSHU cassette (wild-type- I-SceI gene and I-SceI site, hyg marker conferring resistance to hygromycin B, and URA3 gene) within 90-bp direct repeats inside the coding sequence of the LYS2 gene. b lys2::DR I-SceI site contains only the 18-bp I-SceI site within 90-bp direct repeats inside the coding sequence of the LYS2 gene. cPlasmids expressing wild-type, K223I, or D145A I-SceI (pAG7-wild-type-I-SceI, pAG7-K223I, or pAG7-D145A, respectively) were transformed separately into yeast cells to generate different strains. dSingle gene deletions of RAD51 were generated through targeted replacement with the kanMX4 cassette conferring resistance to G418. eStrain SAS-59 and its derivatives are mating type “α” and contain the I-SceI site inside the coding sequence of the TRP5 gene oriented such that an I-SceI SSB is generated on the “Crick” strand. fStrain SAS-193 and its derivatives are mating type “a” and contain the I-SceI site inside the coding sequence of the TRP5 gene oriented such that an I-SceI SSB is generated on the “Crick” strand. gStrain SAS-278 and its derivatives are mating type “α” and contain the I-SceI site inside the coding sequence of the TRP5 gene oriented such that an I-SceI SSB is generated on the “Watson” strand. hStrain FRO-917 and its derivatives contain a 31-bp insert inside the coding sequence of the TRP5 gene. iThe GSHU-wild-type-I-SceI, GSHU-K223I, or GSHU-D145A cassettes were inserted into the genome approximately 10 kb upstream of the trp5 locus along with the I-SceI site such that an I-SceI SSB is generated on the “Watson” strand. jThe GSHU-wild-type-I-SceI, GSHU-K223I, or GSHU-D145A cassettes were inserted into the genome approximately 10 kb upstream of the trp5 locus along with the I-SceI site such that an I-SceI SSB is generated on the “Crick” strand. kThe GSHU-wild-type-I-SceI, GSHU-K223I, or GSHU-D145A cassettes were inserted into the genome approximately 10 kb downstream of the t [file pone.0088840.s003.docx]

**Table S1. Strains used for yeast studies.**

| **Strain Name** | **Relevant Genotype** | **Source** |
| --- | --- | --- |
| FRO-830 | *MAT*α *leu2-3,112 his7-2 ura3*Δ *trp1-289 lys2*::DR[*GSHU*]^a^ | [34] |
| SAS-50 | FRO-1 *lys2*::DR I-SceI site^b^ | This study |
| SAS-74 | SAS-50 + pAG7-wild-type-I-SceI^c^ | This study |
| SAS-75 | SAS-50 + pAG7-wild-type-I-SceI | This study |
| SAS-77 | SAS-50 + pAG7-K223I^c^ | This study |
| SAS-149 | SAS-50 + pAG7-K223I | This study |
| SAS-142 | SAS-50 + pAG7-D145A^c^ | This study |
| SAS-143 | SAS-50 + pAG7-D145A | This study |
| SAS-174 | SAS-50*rad51*Δ::*kanMX4* + pAG7-wild-type-I-SceI^d^ | This study |
| SAS-175 | SAS-50*rad51*Δ::*kanMX4* + pAG7-wild-type-I-SceI | This study |
| SAS-176 | SAS-50*rad51*Δ::*kanMX4* + pAG7-K223I | This study |
| SAS-177 | SAS-50*rad51*Δ::*kanMX4* + pAG7-K223I | This study |
| SAS-178 | SAS-50*rad51*Δ::*kanMX4*+ pAG7-D145A | This study |
| SAS-179 | SAS-50*rad51*Δ::*kanMX4*+ pAG7-D145A | This study |
| FRO-1 | *MAT*α *ade5*-*1 his7*-*2 leu2*-*3*,*112 ura3*-*52 trp5*::*GSHU lys2*::*Alu IR* | [34] |
| SAS-59 | FRO-1 *trp5*::C I-SceI site^e^ | This study |
| SAS-78 | SAS-59 + pAG7-wild-type-I-SceI | This study |
| SAS-79 | SAS-59 + pAG7-wild-type-I-SceI | This study |
| SAS-80 | SAS-59 + pAG7-K223I | This study |
| SAS-148 | SAS-59 + pAG7-K223I | This study |
| SAS-116 | SAS-59 + pAG7-D145A | This study |
| SAS-117 | SAS-59 + pAG7-D145A | This study |
| SAS-182 | SAS-59 *bar1*::*URA3* | This study |
| SAS-193 | SAS-182 *MAT***a**^f^ | This study |
| SAS-227 | SAS-193 + pAG7-wild-type-I-SceI | This study |
| SAS-228 | SAS-193 + pAG7-wild-type-I-SceI | This study |
| SAS-229 | SAS-193 + pAG7-K223I | This study |
| SAS-230 | SAS-193 + pAG7-K223I | This study |
| SAS-231 | SAS-193 + pAG7-D145A | This study |
| SAS-232 | SAS-193 + pAG7-D145A | This study |
| SAS-205 | SAS-193 *rad51*Δ::*kanMX4* | This study |
| SAS-235 | SAS-205 + pAG7-wild-type-I-SceI | This study |
| SAS-236 | SAS-205 + pAG7-wild-type-I-SceI | This study |
| SAS-237 | SAS-205 + pAG7-K223I | This study |
| SAS-238 | SAS-205 + pAG7-K223I | This study |
| SAS-239 | SAS-205 + pAG7-D145A | This study |
| SAS-240 | SAS-205 + pAG7-D145A | This study |
| SAS-278 | FRO-1 *trp5*::W I-SceI site^g^ | This study |
| SAS-281 | SAS-278 + pAG7-wild-type-I-SceI | This study |
| SAS-282 | SAS-278 + pAG7-wild-type-I-SceI | This study |
| SAS-283 | SAS-278 + pAG7-K223I | This study |
| SAS-284 | SAS-278 + pAG7-K223I | This study |
| SAS-285 | SAS-278 + pAG7-D145A | This study |
| SAS-286 | SAS-278 + pAG7-D145A | This study |
| FRO-917 | *MAT*α *his3Δ1 leu2Δ0 lys2Δ0 ura3Δ0 trp5::*INS31^h^ | [34] |
| FRO-879 | *MAT***a** *his3Δ1 leu2Δ0 met15Δ0 ura3Δ0 trp5*::*LEU2* | [34] |
| FRO-872 | FRO-917 GSHU-wild-type I-*Sce*I 10 kb upstream (C I-SceI site)^i^ | [34] |
| SAS-138 | FRO-917 GSHU-K223I 10 kb upstream (C I-SceI site)^i^ | This study |
| SAS-140 | FRO-917 GSHU-D145A 10 kb upstream (C I-SceI site)^i^ | This study |
| SAS-150 | FRO-872 / FRO-879 diploid | This study |
| SAS-151 | FRO-872 / FRO-879 diploid | This study |
| SAS-162 | SAS-138 / FRO-879 diploid | This study |
| SAS-163 | SAS-138 / FRO-879 diploid | This study |
| SAS-166 | SAS-140 / FRO-879 diploid | This study |
| SAS-167 | SAS-140 / FRO-879 diploid | This study |
| SAS-199 | FRO-917 GSHU-wild-type I-SceI 10 kb upstream (W I-SceI site)^j^ | This study |
| SAS-200 | FRO-917 GSHU-wild-type I-SceI 10 kb upstream (W I-SceI site) ^j^ | This study |
| SAS-195 | FRO-917 GSHU-K223I 10 kb upstream (W I-SceI site)^j^ | This study |
| SAS-196 | FRO-917 GSHU-K223I 10 kb upstream (W I-SceI site) | This study |
| SAS-197 | FRO-917 GSHU-D145A 10 kb upstream (W I-SceI site)^j^ | This study |
| SAS-198 | FRO-917 GSHU-D145A 10 kb upstream (W I-SceI site) | This study |
| SAS-215 | SAS-199 / FRO-879 diploid | This study |
| SAS-217 | SAS-200 / FRO-879 diploid | This study |
| SAS-207 | SAS-195 / FRO-879 diploid | This study |
| SAS-209 | SAS-196 / FRO-879 diploid | This study |
| SAS-211 | SAS-197 / FRO-879 diploid | This study |
| SAS-213 | SAS-198 / FRO-879 diploid | This study |
| FRO-876 | FRO-917 GSHU-wild-type I-SceI 10 kb downstream (W I-SceI site)^k^ | [34] |
| SAS-134 | FRO-917 GSHU-K223I 10 kb downstream (W I-SceI site)^k^ | This study |
| SAS-135 | FRO-917 GSHU-K223I 10 kb downstream (W I-SceI site) | This study |
| SAS-136 | FRO-917 GSHU-D145A 10 kb downstream (W I-SceI site)^k^ | This study |
| SAS-137 | FRO-917 GSHU-D145A 10 kb downstream (W I-SceI site) | This study |
| SAS-152 | FRO-876 / FRO-879 diploid | This study |
| SAS-153 | FRO-876 / FRO-879 diploid | This study |
| SAS-154 | SAS-134 / FRO-879 diploid | This study |
| SAS-156 | SAS-135 / FRO-879 diploid | This study |
| SAS-158 | SAS-136 / FRO-879 diploid | This study |
| SAS-160 | SAS-137 / FRO-879 diploid | This study |
| SAS-269 | FRO-917 GSHU-wild-type I-SceI 10 kb downstream (C I-SceI site)^l^ | This study |
| SAS-270 | FRO-917 GSHU-wild-type I-SceI 10 kb downstream (C I-SceI site) | This study |
| SAS-201 | FRO-917 GSHU-K223I 10 kb downstream (C I-SceI site)^l^ | This study |
| SAS-202 | FRO-917 GSHU-K223I 10 kb downstream (C I-SceI site) | This study |
| SAS-245 | FRO-917 GSHU-D145A 10 kb downstream (C I-SceI site)^l^ | This study |
| SAS-246 | FRO-917 GSHU-D145A 10 kb downstream (C I-SceI site) | This study |
| SAS-272 | SAS-269 / FRO-879 diploid | This study |
| SAS-274 | SAS-270 / FRO-879 diploid | This study |
| SAS-219 | SAS-201 / FRO-879 diploid | This study |
| SAS-221 | SAS-202 / FRO-879 diploid | This study |
| SAS-251 | SAS-245 / FRO-879 diploid | This study |
| SAS-253 | SAS-246 / FRO-879 diploid | This study |

^a^*lys2*::DR[GSHU] contains the GSHU cassette (wild-type- I-SceI gene and I-SceI site, *hyg* marker conferring resistance to hygromycin B, and *URA3* gene) within 90-bp direct repeats inside the coding sequence of the *LYS2* gene

^b^*lys2*::DR I-SceI site contains only the 18-bp I-SceI site within 90-bp direct repeats inside the coding sequence of the *LYS2* gene

^c^Plasmids expressing wild-type, K223I, or D145A I-SceI (pAG7-wild-type-I-SceI, pAG7-K223I, or pAG7-D145A, respectively) were transformed separately into yeast cells to generate different strains

^d^Single gene deletions of *RAD51* were generated through targeted replacement with the *kanMX4* cassette conferring resistance to G418

^e^Strain SAS-59 and its derivatives are mating type “α” and contain the I-SceI site inside the coding sequence of the *TRP5* gene oriented such that an I-SceI SSB is generated on the “Crick” strand

^f^Strain SAS-193 and its derivatives are mating type “**a**” and contain the I-SceI site inside the coding sequence of the *TRP5* gene oriented such that an I-SceI SSB is generated on the “Crick” strand

^g^Strain SAS-278 and its derivatives are mating type “α” and contain the I-SceI site inside the coding sequence of the *TRP5* gene oriented such that an I-SceI SSB is generated on the “Watson” strand

^h^Strain FRO-917 and its derivatives contain a 31-bp insert inside the coding sequence of the *TRP5* gene

^i^The GSHU-wild-type-I-SceI, GSHU-K223I, or GSHU-D145A cassettes were inserted into the genome approximately 10 kb upstream of the *trp5* locus along with the I-SceI site such that an I-SceI SSB is generated on the “Watson” strand

^j^The GSHU-wild-type-I-SceI, GSHU-K223I, or GSHU-D145A cassettes were inserted into the genome approximately 10 kb upstream of the *trp5* locus along with the I-SceI site such that an I-SceI SSB is generated on the “Crick” strand

^k^The GSHU-wild-type-I-SceI, GSHU-K223I, or GSHU-D145A cassettes were inserted into the genome approximately 10 kb downstream of the *trp5* locus along with the I-SceI site such that an I-SceI SSB is generated on the “Watson” strand

^l^The GSHU-wild-type-I-SceI, GSHU-K223I, or GSHU-D145A cassettes were inserted into the genome approximately 10 kb downstream of the *trp5* locus along with the I-SceI site such that an I-SceI SSB is generated on the “Crick” strand
