# Supplementary material for: To Nick or Not to Nick: Comparison of I-SceI Single- and Double-Strand Break-Induced Recombination in Yeast and Human Cells
Source: PLoS One. 2014 Feb 18;9(2):e88840. doi: 10.1371/journal.pone.0088840 (PMC3928301; doi:10.1371/journal.pone.0088840)
Supplement: Table S2 — Oligos used for repair assays. The sequence of the oligos used for repair of the disrupted trp5 (yeast), GFP (human cells), or DsRed2 (human cells) loci are listed from the 5′ ends of each 80-base sequence. (DOCX) [file pone.0088840.s004.docx]

**Table S2. Oligos used for repair assays.**

| **Gene** | **Oligo** | **Size** | **Sequence** |
| --- | --- | --- | --- |
| *TRP5* | TRP5.80F | 80-mer | 5’ – GTCTAAGAGAGTTGGAAAAGGGTTTTGATGAAGCTGTCGCGGATCCCACATT  CTGGGAAGACTTCAAATCCTTGTATTCT |
| *TRP5* | TRP5.80R | 80-mer | 5’ – AGAATACAAGGATTTGAAGTCTTCCCAGAATGTGGGATCCGCGACAGCTTCAT  CAAAACCCTTTTCCAACTCTCTTAGAC |
| GFP | GFP80.F | 80-mer | 5’ – GCGCACCATCTTCTTCAAGGACGACGGCAACTACAAGACGCGCGCCGAGGTG  AAGTTCGAGGGCGACACCCTGGTGAACC |
| GFP | GFP80.R | 80-mer | 5’ – GGTTCACCAGGGTGTCGCCCTCGAACTTCACCTCGGCGCGCGTCTTGTAGTTG  CCGTCGTCCTTGAAGAAGATGGTGCGC |
| DsRed2 | DsRed2.80F | 80-mer | 5’ – GGCGGCGTGGCGACCGTGACCCAGGACTCCTCCCTGCAGGACGGCTGCTTCA  TCTACAAGGTGAAGTTCATCGGCGTGAA |
| DsRed2 | DsRed2.80R | 80-mer | 5’ – TTCACGCCGATGAACTTCACCTTGTAGATGAAGCAGCCGTCCTGCAGGGAGG  AGTCCTGGGTCACGGTCGCCACGCCGCC |

The sequence of the oligos used for repair of the disrupted *trp5* (yeast), GFP (human cells), or DsRed2 (human cells) loci are listed from the 5’ ends of each 80-base sequence.
