# Supplementary material for: To Nick or Not to Nick: Comparison of I-SceI Single- and Double-Strand Break-Induced Recombination in Yeast and Human Cells
Source: PLoS One. 2014 Feb 18;9(2):e88840. doi: 10.1371/journal.pone.0088840 (PMC3928301; doi:10.1371/journal.pone.0088840)
Supplement: Table S3 — Supplementary data for graphs. Data from Figure 2B. Data are presented as the median with the range in parentheses; n≥11. Results were statistically analyzed using the Mann-Whitney U test. aStrains used: RAD51 (SAS-74 and SAS-75) and rad51Δ (SAS-174 and SAS-175). bStrains used: RAD51 (SAS-77 and SAS-149) and rad51Δ (SAS-176 and SAS-177). cStrains used: RAD51 (SAS-142 and SAS-143) and rad51Δ (SAS-178 and SAS-179). ** p≤0.01, **** p≤0.0001, NS = not significant, n/a (not applicable). (DOCX) [file pone.0088840.s005.docx]

**Table S3. Supplementary data for graphs.**

**A**

|  | **No. of Lys^+^ colonies per 10^7^ viable cells** | | | | |
| --- | --- | --- | --- | --- | --- |
| **Protein** | ***RAD51*** | ***rad51*Δ** | **Fold change in *RAD51* background**  **(Wild-type or K223I / D145A)** | **Fold change in *rad51* background**  **(Wild-type or K223I / D145A)** | **Fold change**  **(*rad51Δ I RAD51*)** |
| **Wild-type I-SceI^a^** | 1,230,000  (1,067,000 – 1,480,000) | 1,800,000  (1,510,000 – 2,570,000) | 2,420**** | 2,020**** | 1.46**** |
| **K223I I-SceI^b^** | 1,240  (891 – 1,800) | 1,413  (857 – 2,070) | 2.44**** | 1.58** | NS  (p = 0.4302) |
| **D145A I-SceI^c^** | 509  (360 - 805) | 893  (583 – 1,030) | n/a | n/a | 1.75**** |

Data from Figure 2B. Data are presented as the median with the range in parentheses; n≥11. Results were statistically analyzed using the Mann-Whitney U test.

^a^Strains used: *RAD51* (SAS-74 and SAS-75) and *rad51*Δ (SAS-174 and SAS-175)

^b^Strains used: *RAD51* (SAS-77 and SAS-149) and *rad51*Δ (SAS-176 and SAS-177)

^c^Strains used: *RAD51* (SAS-142 and SAS-143) and *rad51*Δ (SAS-178 and SAS-179)

** p≤ 0.01, **** p ≤ 0.0001, NS = not significant, n/a (not applicable)

**B**

|  |  | **No. of Trp^+^ colonies per 10^7^ viable cells** | | | | | | | |
| --- | --- | --- | --- | --- | --- | --- | --- | --- | --- |
|  |  | **Strand of K223I cleavage** | |  | **Fold change in *RAD51* background**  **(Wild-type or K223I / D145A)^d^** | |  | **Single strand oligo preference (F/R)** | |
| **Protein** | **Oligo** | **“Crick”** | **“Watson”** |  | **“Crick”** | **“Watson”** |  | **“Crick”** | **“Watson”** |
| **Wild-type I-SceI^a^** | **No oligo** | 33.6  (18.3 – 48.9) | 1.15  (0.85 – 1.45) |  | n/a | n/a |  |  |  |
|  | **F** | 363  (211 – 433) | 216  (150 – 354) |  | 192** | 220** |  | NS  (p = 0.4848) | NS  (p = 1.000) |
|  | **R** | 298  (248 – 416) | 301  (119 – 362) |  | 82.8** | 149** |  |  |  |
|  | **Pair** | 1,440  (1,290 – 1,740) | 2,200  (1,790 – 2,810) |  | 159** | 345** |  |  |  |
| **K223I I-SceI^b^** | **No oligo** | <1  (0 – 0) | <1  (0 – 0) |  | n/a | n/a |  |  |  |
|  | **F** | 15.5  (6 – 34.4) | 8.06  (6.10 – 13.1) |  | 9.04** | 8.24** |  | NS  (p = 0.1797) | NS  (p = 0.3776) |
|  | **R** | 26.1  (11.7 – 44) | 11.1  (1.83 – 22.7) |  | 8.2** | 5.53** |  |  |  |
|  | **Pair** | 39.7  (17.4 – 69.3) | 20.2  (13.2 – 38.6) |  | 4.51** | 3.16** |  |  |  |
| **D145A I-SceI^c^** | **No oligo** | 0.11  (0 – 0.21) | <1  (0 – 0) |  | n/a | n/a |  |  |  |
|  | **F** | 1.74  (0.68 – 4.62) | 0.98  (0.43 – 3.91) |  | n/a | n/a |  | NS  (p = 0.3939) | NS  (p = 0.3776) |
|  | **R** | 3.29  (0.34 – 6.93) | 2.01  (0.14 – 3.75) |  | n/a | n/a |  |  |  |
|  | **Pair** | 8.91  (1.71 – 20.8) | 6.39  (3.28 – 8.5) |  | n/a | n/a |  |  |  |

Data from Figure 3B. Data are presented as the median with the range in parentheses; n=6. Results were statistically analyzed using the Mann-Whitney U test.

^a^Strains used: “Crick” (SAS-227 and SAS-228) and “Watson” (SAS-281 and SAS-282)

^b^Strains used: “Crick” (SAS-229 and SAS-230) and “Watson” (SAS-283 and SAS-284)

^c^Strains used: “Crick” (SAS-231 and SAS-232) and “Watson” (SAS-285 and SAS-286)

^d^Background values (no oligo) were subtracted prior to statistical analysis

** p< 0.01, NS = not significant, n/a (not applicable)

**C**

|  |  | **No. of Trp^+^ colonies per 10^7^ viable cells** | | | | |  |
| --- | --- | --- | --- | --- | --- | --- | --- |
| **Protein** | **Oligo** | ***RAD51*** | ***rad51Δ*** | **Fold change in *RAD51* background**  **(Wild-type or K223I / D145A)^d^** | **Fold change in *rad51* background**  **(Wild-type or K223I / D145A)^d^** | **Fold change**  **(*rad51Δ I RAD51*)^d^** | |
| **Wild-type I-SceI^a^** | **No oligo** | 13.2  (8.89 – 17.6) | 26.5  (21.7 – 31.3) | n/a | n/a | n/a | |
|  | **F** | 63.4  (38.0 – 92.7) | 276  (146 – 323) | 11.2** | 1,340** | 4.89** | |
|  | **R** | 71.9  (56.9 – 115) | 197  (140 – 239) | 10.1** | 1,700** | 2.9** | |
|  | **Pair** | 622  (502 – 1,070) | 5,010  (2,300 – 6,280) | 35.8** | 1,810** | 8.12** | |
| **K223I I-SceI^b^** | **No oligo** | <1  (0 – 0) | <1  (0 – 0) | n/a | n/a | n/a | |
|  | **F** | 7.61  (3.81 – 12.1) | 0.22  (<0.1 – 1.35) | 1.71* | NS  (p = 0.4805) | 0.03** | |
|  | **R** | 11.9  (8.25 – 14.4) | 0.45  (<0.1 – 0.9) | 2.05** | NS  (p = 0.0606) | 0.04** | |
|  | **Pair** | 22.7  (20.0 – 27.6) | 2.49  (0.90 – 6.28) | 1.32* | NS  (p > 0.9999) | 0.11** | |
| **D145A I-SceI^c^** | **No oligo** | <1  (0 – 0) | <1  (0 – 0) | n/a | n/a | n/a | |
|  | **F** | 4.46  (2.86 – 5.19) | 0.18  (<0.1 – 0.38) | n/a | n/a | 0.04** | |
|  | **R** | 5.80  (4.13 – 10) | <1  (<0.1 – 0.38) | n/a | n/a | 0.02** | |
|  | **Pair** | 17.1  (13.5 – 22.2) | 2.75  (0.76 – 4.03) | n/a | n/a | 0.16** | |

Data from Figure 3C. Data are presented as the median with the range in parentheses; n≥5. Results were statistically analyzed using the Mann-Whitney U test.

^a^Strains used: *RAD51* (SAS-227 and SAS-228) and *rad51*Δ (SAS-235 and SAS-236)

^b^Strains used: *RAD51* (SAS-229 and SAS-230) and *rad51*Δ (SAS-237 and SAS-238)

^c^Strains used: *RAD51* (SAS-231 and SAS-232) and *rad51*Δ (SAS-239 and SAS-240)

^d^Background values (no oligo) were subtracted prior to statistical analysis

* p< 0.05, ** p< 0.01, n/a (not applicable)

**D**

|  |  | **No. of Trp^+^ colonies per 10^7^ viable cells** | | | | |  |
| --- | --- | --- | --- | --- | --- | --- | --- |
| **Protein** | **Oligo** | ***RAD51*** | ***rad51Δ*** | **Fold change in *RAD51* background**  **(Wild-type / D145A)^c^** | **Fold change in *rad51* background**  **(Wild-type / D145A)^c^** | **Fold change**  **(*rad51Δ I RAD51*)^c^** | |
| **Wild-type I-SceI^a^**  **(with 2% galactose)** | **No oligo** | 44.6  (19.4 – 99) | 73.1  (34.7 – 217) | n/a | n/a | n/a | |
|  | **F** | 132  (106 – 179) | 434  (297 – 540) | 24.2* | 2,700** | 5.49* | |
|  | **R** | 136  (103 – 199) | 403  (313 – 569) | 10.5* | 2,390** | 4.61* | |
|  | **Pair** | 592  (504 – 667) | 6,060  (4,890 – 7,500) | 151** | 6,070** | 10.5** | |
| **Wild-type I-SceI^a^**  **(with 0.02% galactose)** | **No oligo** | 30.6  (10.7 – 65.9) | 91.6  (25.4 – 161) | n/a | n/a | n/a | |
|  | **F** | 77.2  (51.4 – 113) | 223  (181 – 318) | 10.2** | 750** | 3.64* | |
|  | **R** | 77.4  (54.9 – 136) | 204  (194 – 225) | 4.24** | 611** | 2.92* | |
|  | **Pair** | 218  (151 – 365) | 1,430  (1,190 – 1,770) | 54.2** | 1,400** | 6.75** | |
| **D145A I-SceI^b^**  **(with 2% galactose)** | **No oligo** | <1  (0 – 0) | <1  (0 – 0) | n/a | n/a | n/a | |
|  | **F** | 2.02  (1.28 – 6.34) | <1  (<1 – 3.57) | n/a | n/a | 0.05* | |
|  | **R** | 4.93  (0.64 – 5.77) | <1  (0 – 0) | n/a | n/a | 0.02* | |
|  | **Pair** | 3.80  (2.24 – 4.85) | 0.99  (0 – 1.76) | n/a | n/a | 0.26* | |

Data from Figure 3D. Data are presented as the median with the range in parentheses; n=6. Results were statistically analyzed using the Mann-Whitney U test.

^a^Strains used: *RAD51* (SAS-227 and SAS-228) and *rad51*Δ (SAS-235 and SAS-236)

^b^Strains used: *RAD51* (SAS-231 and SAS-232) and *rad51*Δ (SAS-239 and SAS-240)

^c^Background values (no oligo) were subtracted prior to statistical analysis

* p< 0.05, ** p< 0.01, n/a (not applicable)

**E**

|  |  | **No. of Trp^+^ colonies per 10^7^ viable cells** | | |
| --- | --- | --- | --- | --- |
| **Protein** | **Oligo** | **Cycling** | **G1** | **Fold change**  **(cycling/G1)^d^** |
| **Wild-type I-SceI^a^** | **No oligo** | 27.7  (22.8 – 32.6) | 14.9  (12.7 – 17.2) | n/a |
|  | **F** | 292  (161 – 731) | 66.7  (47.9 – 113) | 4.3** |
|  | **R** | 240  (138 – 577) | 91.9  (66.2 – 139) | 2.6** |
|  | **Pair** | 2,270  (1,900 – 2,620) | 302  (257 – 371) | 7.5** |
| **K223I I-SceI^b^** | **No oligo** | <1  (0 – 0) | <1  (0 – 0) | n/a |
|  | **F** | 16.9  (12.1 – 27.5) | 21.0  (11.1 – 92.6) | NS  (p = 0.5745) |
|  | **R** | 28.9  (21.1 – 42.2) | 26.5  (14.8 – 47.1) | NS  (p = 0.5887) |
|  | **Pair** | 38.1  (27.1 – 69.7) | 23.5  (11.8 – 40.7) | NS  (p = 0.0649) |
| **D145A I-SceI^c^** | **No oligo** | <1  (0 – 0.23) | <1  (0 – 0) | n/a |
|  | **F** | 2.08  (1.41 – 4.71) | 4.41  (0 – 10.5) | NS  (p = 0.4696) |
|  | **R** | 7.13  (3.29 – 9.06) | 8.82  (2.63 – 10.5) | NS  (p = 0.5887) |
|  | **Pair** | 9.42  (3.29 – 13.8) | 13.2  (0 – 31.6) | NS  (p = 0.2971) |

Data from Figure 4. Data are presented as the median with the range in parentheses; n≥5. Results were statistically analyzed using the Mann-Whitney U test.

^a^Strains used: SAS-227 and SAS-228

^b^Strains used: SAS-229 and SAS-230

^c^Strains used: SAS-231 and SAS-232

^d^Background values (no oligo) were subtracted prior to statistical analysis

** p< 0.01, NS = not significant, n/a (not applicable)

**F**

|  |  | **No. of fluorescent cells detected per 100,000 cells read** | | |  |
| --- | --- | --- | --- | --- | --- |
| **Protein** | **Oligo** |  | **Fold increase**  **(Wild-type or K223I / D145A)** | **Single strand oligo preference** | |
| **Wild-type I-SceI** | **F** | 66.5  (55 – 106) | 26.6** | NS  (p = 0.6991) | |
|  | **R** | 77.0  (35 – 94) | 38.5** |  |  |
| **K223I I-SceI** | **F** | 8  (2 – 10) | NS  (p = 0.1215) | NS  (p = 0.3743) | |
|  | **R** | 4  (3 – 5) | 2* |  |  |
| **D145A I-SceI** | **F** | 2.5  (1 – 5) | n/a | NS  (p = 0.3593) | |
|  | **R** | 2  (0 – 5) | n/a |  |  |

Data from Figure 5B. Data are presented as the median with the range in parentheses; n=6. Results were statistically analyzed using the Mann-Whitney U test.

* p<0.05, ** p< 0.01, NS = not significant, n/a (not applicable)

**G**

|  |  | **No. of fluorescent cells detected per 100,000 cells read** | | |  |
| --- | --- | --- | --- | --- | --- |
| **Protein** | **Oligo** |  | **Fold increase**  **(Wild-type or K223I / D145A)** | **Single strand oligo preference** | |
| **Wild-type I-SceI** | **F** | 82  (51 – 89) | 674**** | ****  in favor of F | |
|  | **R** | 25  (7 – 38) | 228**** |  |  |
| **K223I I-SceI** | **F** | <1  (0 – 1) | NS  (p > 0.9999) | **  in favor of R | |
|  | **R** | 1  (0 – 3) | 12** |  |  |
| **D145A I-SceI** | **F** | <1  (0 – 1) | n/a | NS  (p > 0.9999) | |
|  | **R** | <1  (0 – 1) | n/a |  |  |

Data from Figure 5C. Data are presented as the median with the range in parentheses; n=9. Results were statistically analyzed using the Mann-Whitney U test.

** p< 0.01,**** p ≤ 0.0001,NS = not significant, n/a (not applicable)

**H**

|  |  | **No. of fluorescent cells detected per 100,000 cells read** | | |  |
| --- | --- | --- | --- | --- | --- |
| **Protein** | **Oligo** |  | **Fold increase**  **(Wild-type or K223I / D145A)** | **Single strand oligo preference** | |
| **Wild-type I-SceI** | **F** | 38.5  (22 – 70) | 330**** | ****  in favor of F | |
|  | **R** | 9  (4 – 12) | 77*** |  |  |
| **K223I I-SceI** | **F** | 0.5  (0 – 1) | NS  (p = 0.0769) | *  in favor of R | |
|  | **R** | 1  (0 – 2) | 12*** |  |  |
| **D145A I-SceI** | **F** | <1  (0 – 0) | n/a | NS  (p > 0.9999) | |
|  | **R** | <1  (0 – 0) | n/a |  |  |

Data from Figure 5D. Data are presented as the median with the range in parentheses; n≥8. Results were statistically analyzed using the Mann-Whitney U test.

* p<0.05, *** p< 0.001, **** p ≤ 0.0001, NS = not significant, n/a (not applicable)

**I**

|  |  |  | **No. of Trp^+^ colonies per 10^7^ viable cells** | | | | | | | | | | |
| --- | --- | --- | --- | --- | --- | --- | --- | --- | --- | --- | --- | --- | --- |
|  |  |  | **Strand of K223I cleavage** | |  | **Fold change**  **(Wild-type or K223I / D145A)^d^** | | |  | | **Single strand oligo preference** | | |
| **Protein** | **Oligo** |  | **“Crick”** | **“Watson”** |  | **“Crick”** | **“Watson”** |  | | **“Crick”** | | **“Watson”** |  |
| **Wild-type I-SceI^a^** | **No oligo** |  | <1  (0 – 0) | <1  (0 – 0) |  | n/a | n/a |  | |  | |  |  |
|  | **F** |  | 134  (77.3 – 186) | 31.1  (22.4 – 39.1) |  | 9.64* | 7.13* |  | | *  in favor of R | | *  in favor of R |  |
|  | **R** |  | 592  (378 – 898) | 97.4  (84.2 – 153) |  | 35.2* | 20.2* |  | |  |  |  |  |
|  | **Pair** |  | 1,190  (874 – 1,650) | 133  (112 – 218) |  | 30.4* | 10.6* |  | |  | |  |  |
| **K223I I-SceI^b^** | **No oligo** |  | <1  (0 – 0) | <1  (0 – 0) |  | n/a | n/a |  | |  | |  |  |
|  | **F** |  | 22.3  (18.7 – 27.3) | 15.3  (10.4 – 17.9) |  | NS  (p = 0.0571) | 3.51* |  | | NS  (p = 0.1143) | | NS  (p = 0.0571) |  |
|  | **R** |  | 29.1  (21.3 – 31.6) | 24.8  (17.8 – 31.1) |  | 1.73* | 5.16* |  | |  |  |  |  |
|  | **Pair** |  | 89.2  (54.5 – 129) | 53.0  (40.2 – 67.1) |  | 2.28* | 4.21* |  | |  | |  |  |
| **D145A I-SceI^c^** | **No oligo** |  | <1  (0 – 0) | <1  (0 – 0) |  | n/a | n/a |  | |  | |  |  |
|  | **F** |  | 13.9  (9.18 – 20) | 4.36  (2.96 – 7.41) |  | n/a | n/a |  | | NS  (p = 0.5614) | | NS  (p = 1.0000) |  |
|  | **R** |  | 16.8  (12.8 – 20) | 4.81  (3.70 – 5.48) |  | n/a | n/a |  | |  |  |  |  |
|  | **Pair** |  | 39.1  (38.2 – 51) | 12.6  (7.57 – 14.1) |  | n/a | n/a |  | |  | |  |  |

Data from Figure 6A. Data are presented as the median with the range in parentheses; n=4. Results were statistically analyzed using the Mann-Whitney U test.

^a^Strains used: “Crick” (SAS-150 and SAS-151) and “Watson” (SAS-215 and SAS-217)

^b^Strains used: “Crick” (SAS-162 and SAS-163) and “Watson” (SAS-207 and SAS-209)

^c^Strains used: “Crick” (SAS-166 and SAS-167) and “Watson” (SAS-211 and SAS-213)

^d^Background values (no oligo) were subtracted prior to statistical analysis

*p< 0.05, NS = not significant, n/a (not applicable)

**J**

|  |  |  | **No. of Trp^+^ colonies per 10^7^ viable cells** | | | | | | | | | | | | |
| --- | --- | --- | --- | --- | --- | --- | --- | --- | --- | --- | --- | --- | --- | --- | --- |
|  |  |  | **Strand of K223I cleavage** | |  | | **Fold change**  **(Wild-type or K223I / D145A)^d^** | | |  | | **Single strand oligo preference** | | |  |
| **Protein** | **Oligo** |  | **“Watson”** | **“Crick”** | |  | | **“Watson”** | **“Crick”** | |  | | **“Watson”** | **“Crick”** | |
| **Wild-type I-SceI^a^** | **No oligo** |  | <1  (0 – 0) | <1  (0 – 0) | |  | | n/a | n/a | |  | |  |  | |
|  | **F** |  | 407  (332 – 475) | 139  (117 – 195) | |  | | 17.1* | 10.5* | |  | | *  in favor of F | *  in favor of F | |
|  | **R** |  | 161  (127 – 176) | 88.5  (76.0 – 106) | |  | | 5.08* | 5.33* | |  | |  |  |  |
|  | **Pair** |  | 405  (309 – 455) | 203  (155 – 251) | |  | | 15.4* | 14.9* | |  | |  |  | |
| **K223I I-SceI^b^** | **No oligo** |  | <1  (0 – 0) | <1  (0 – 0) | |  | | n/a | n/a | |  | |  |  | |
|  | **F** |  | 66.8  (47.2 – 101) | 20.4  (13.4 – 22.8) | |  | | 2.81* | NS  (p = 0.1143) | |  | | NS  (p = 0.4857) | NS  (p = 0.6857) | |
|  | **R** |  | 73.2  (64.3 – 130) | 25.1  (16.0 – 38.1) | |  | | 2.31* | NS  (p = 0.1143) | |  | |  |  |  |
|  | **Pair** |  | 61.0  (49.5 – 71.6) | 20.0  (14.4 – 29) | |  | | 2.32* | NS  (p = 0.0571) | |  | |  |  | |
| **D145A I-SceI^c^** | **No oligo** |  | <1  (0 – 0) | <1  (0 – 0) | |  | | n/a | n/a | |  | |  |  | |
|  | **F** |  | 23.8  (11.8 – 37.6) | 13.3  (12.4 – 15.5) | |  | | n/a | n/a | |  | | NS  (p = 0.8867) | NS  (p = 0.0571) | |
|  | **R** |  | 31.7  (15.9 – 46.8) | 16.6  (14.6 – 19) | |  | | n/a | n/a | |  | |  |  |  |
|  | **Pair** |  | 26.3  (22.3 – 43.7) | 13.6  (12.4 – 14.5) | |  | | n/a | n/a | |  | |  |  | |

Data from Figure 6B. Data are presented as the median with the range in parentheses; n=4. Results were statistically analyzed using the Mann-Whitney U test.

^a^Strains used: “Watson” (SAS-152 and SAS-153) and “Crick” (SAS-272 and SAS-274)

^b^Strains used: “Watson” (SAS-154 and SAS-156) and “Crick” (SAS-219 and SAS-221)

^c^Strains used: “Watson” (SAS-158 and SAS-160) and “Crick” (SAS-251 and SAS-253)

^d^Background values (no oligo) were subtracted prior to statistical analysis

* p< 0.05, NS = not significant, n/a (not applicable)

**K**

|  |  | **No. of fluorescent cells detected per 100,000 cells read** | | |  |
| --- | --- | --- | --- | --- | --- |
| **Protein** | **Oligo** |  | **Fold increase**  **(Wild-type or K223I / D145A)** | **Single strand oligo preference** | |
| **Wild-type I-SceI** | **F** | 7  (1 – 16) | 9.6  * | *  in favor of F | |
|  | **R** | 2.50  (0 – 10) | NS  (p = 0.0906) |  |  |
| **K223I I-SceI** | **F** | 1.5  (0 – 5) | NS  (p = 0.2731) | NS  (p > 0.9999) | |
|  | **R** | 0.50  (0 – 3) | NS  (p = 0.6628) |  |  |
| **D145A I-SceI** | **F** | 1.00  (0 – 2) | n/a | NS  (p > 0.9999) | |
|  | **R** | 0.5  (0 – 1) | n/a |  |  |

Data from Figure 7. Data are presented as the median with the range in parentheses; n=6. Results were statistically analyzed using the Mann-Whitney U test.

* p<0.05, *** p< 0.001, **** p ≤ 0.0001, NS = not significant, n/a (not applicable)
